# Mitochondrial Dysfunction by FADDosome Promotes Gastric Mucosal Injury in Portal Hypertensive Gastropathy

**DOI:** 10.7150/ijbs.90835

**Published:** 2024-04-29

**Authors:** Yuelin Xiao, Yiwang Zhang, Kaiduan Xie, Xiaoli Huang, Xianzhi Liu, Jinni Luo, Siwei Tan

**Affiliations:** 1 Department of Gastroenterology, the Third Affiliated Hospital of Sun Yat-sen University, Guangzhou, Guangdong Province, 510630, China.; 2 Department of Pathology, the Third Affiliated Hospital of Sun Yat-sen University, Guangzhou, Guangdong Province, 510630, China.

**Keywords:** portal hypertensive gastropathy, FADDosome, mitochondrial dysfunction, NLRP3 inflammasome, pyroptosis.

## Abstract

Mucosal epithelial death is an essential pathological characteristic of portal hypertensive gastropathy (PHG). FADDosome can regulate mucosal homeostasis by controlling mitochondrial status and cell death. However, it remains ill-defined whether and how the FADDosome is involved in the epithelial death of PHG. The FADDosome formation, mitochondrial dysfunction, glycolysis process and NLRP3 inflammasome activation in PHG from both human sections and mouse models were investigated. *NLRP3* wild-type (*NLRP3*-WT) and *NLRP3* knockout (*NLRP3*-KO) littermate models, critical element inhibitors and cell experiments were utilized. The mechanism underlying FADDosome-regulated mitochondrial dysfunction and epithelial death in PHG was explored. Here, we found that FADD recruited caspase-8 and receptor-interacting serine/threonine-protein kinase 1 (RIPK1) to form the FADDosome to promote Drp1-dependent mitochondrial fission and dysfunction in PHG. Also, FADDosome modulated NOX2 signaling to strengthen Drp1-dependent mitochondrial fission and alter glycolysis as well as enhance mitochondrial reactive oxygen species (mtROS) production. Moreover, due to the dysfunction of electron transport chain (ETC) and alteration of antioxidant enzymes activity, this altered glycolysis also contributed to mtROS production. Subsequently, the enhanced mtROS production induced NLRP3 inflammasome activation to result in the epithelial pyroptosis and mucosal injury in PHG. Thus, the FADDosome-regulated pathways may provide a potential therapeutic target for PHG.

## Introduction

Portal hypertension (PHT) is one of the most disastrous conditions related to chronic hepatic diseases and always induces gastroesophageal varices (GEVs) and portal hypertensive gastropathy (PHG), which can result in severe gastrointestinal hemorrhages and lead to high morbidity and mortality 1-3. Our previous studies showed that PHT triggers death receptors (DRs) signals, such as TNF-α/TNF receptor 1 (TNFR1) and Fas ligand (FasL)/Fas, contribute to gastric mucosal damage in PHG 4, 5. Fas-associated protein with death domain (FADD) can be recruited by DRs to bind caspase-8 6, 7. The formed FADD and caspase-8 complex is dissociated from the ligated receptor and recruits receptor-interacting serine/threonine-protein kinase 1 (RIPK1) 6-9, which is deubiquitinated and released from the prosurvival complex into this cell death-inducing complex to support cellular death signaling 10, 11, this formation of the FADD/caspase-8/RIPK1 complex is called the FADDosome. Moreover, the FADD/caspase-8 complex can regulate gut homeostasis and inflammation by controlling the death of intestinal epithelial cells 12. The recruitment of RIPK1 by TNF-α to the FADD/caspase-8 complex leads to mitochondrial damage and the cleavage of downstream caspases 13, 14. RIPK1 can phosphorylate dynamin-related protein 1 (Drp1) at serine 616 (Ser616) to enhance Drp1-mediated mitochondrial fission 15. Mitochondria are highly dynamic organelles characterized by a high frequency of fission and fusion events 16, whereas disruption of mitochondrial fission and fusion leads to mitochondrial heterogeneity and dysfunction 17, 18. Drp1, a key component of the mitochondrial division machinery 19, 20, translocates from the cytoplasm to the mitochondrial surface under various stimuli where it interacts with abundant binding partners, including fission protein 1 (Fis1), mitochondrial fission factor (Mff), mitochondrial elongation factor (Mef), mitochondrial dynamics protein 49 (Mid49) and mitochondrial dynamics protein 51 (Mid51), to mediate mitochondrial fission 21, 22. Excessive mitochondrial fission leads to a decrease in the mitochondrial membrane potential, mitochondrial dysfunction, and an increase in mitochondrial reactive oxygen species (mtROS) production, consequently inducing host cell death 23, 24. Mitochondrial dysfunction-induced mtROS have been shown to activate multiple pathways involved in the inflammatory response, NLRP3 activation, dysregulated calcium homeostasis and cell injury in many reversible regulatory processes 25. NLRP3 activation is most closely associated with the initiation of pyroptosis, and NLRP3 recruits and binds to apoptosis-associated speck-like protein (ASC) complexes, which then recruit procaspase-1 to lead to autocleavage and activation of caspase-1. Cleaved caspase-1 is involved in the cleavage and maturation of pro-IL-18 and pro-IL-1β and in the cleavage of gasdermin D (GSDMD), and the N-terminal fragment of GSDMD (GSDMD-N) from the cleavage of GSDMD can ultimately form pores in the plasma membrane, resulting in the secretion of IL-18 and IL-1β and the subsequent cell swelling and pyroptosis 26.

The nicotinamide adenine dinucleotide phosphate (NADPH) oxidase (NOX) family is considered another major source of ROS in eukaryotic cells 27, and NOX elements interact with many canonical signals, such as nuclear factor-κB p65 (NF-κBp65) or mitogen-activated protein kinases (MAPKs), to catalyze the formation of mitochondrial superoxide anion radicals and the downstream generation of hydrogen peroxide 28. The presence of mitochondrial damage or dysfunction, ROS and other factors activate the NLRP3 inflammasome 29, 30, and inappropriate activation of the NLRP3 inflammasome contributes to the initiation of pyroptosis and the onset and progression of a variety of human diseases 31. ROS generated after mitochondrial injury and oxidized mitochondrial DNA (ox-mtDNA) are released into the cytosol, where they lead to NLRP3 inflammasome activation 23. However, the role of mitochondrial dysfunction and mtROS in NLRP3 inflammasome activation-mediated pyroptosis in gastrointestinal diseases is unclear.

In the present study, we verified that portal hypertension induces FADDosome formation on the epithelial mitochondrial membrane to enhance Drp1-dependent mitochondrial fission and dysfunction and alter glycolysis profile; the FADDosome also promotes transforming growth factor-β-activated kinase 1 (TAK1)/NF-κBp65/NOX2 signaling to strengthen Drp1-dependent mitochondrial fission, alter glycolysis process and enhance mtROS production; consequently, the enhanced mtROS generation triggers NLRP3 inflammasome activation and results in gastric epithelial pyroptosis and mucosal injury in PHG. Thus, this network provides a potential therapeutic target for PHG.

## Materials and methods

### Patient tissue samples

Gastric mucosal specimens from 16 PHG patients (for PHG, 8 with hepatitis B virus (HBV)-infected liver cirrhosis and 8 with portal vein occlusion [from the cavernous transformation of the portal vein or portal vein thrombosis without cirrhosis]) without *Helicobacter pylori* (HP) infection who had not yet undergone any therapeutic intervention) and 16 unrelated healthy volunteers (as Uninvolved) who were undergoing a regular healthy physical examination were obtained from the Endoscopic Center of the Third Affiliated Hospital of Sun Yat-sen University. The classification of PHG was based on the Tanoue's three-category system. Informed consent was obtained from all the patients and volunteers prior to inclusion in the study and collection of tissues, and this study protocol was approved by the Institute Research Ethics Committee of the Third Affiliated Hospitals of Sun Yat-sen University (NO. [2021]02-176).

### Mouse models

All animal experimental studies were approved by the Institutional Animal Care and Use Committee of South China Agricultural University (NO. 2022F122). *NLRP3* wild-type (*NLRP3*-WT) and *NLRP3* knockout (*NLRP3*-KO) mice on a C57BL/6 background were purchased from GemPharmatech (NanJing, China). These transgenic mice were identified by genotyping PCR and backcrossed to C57BL/6 wild-type mice. The mice used in this study were housed in a specific pathogen-free (SPF) environment maintained at constant temperature (22 ± 2 °C) and humidity (50 - 60%) with a 12-h light cycle. Mice were kept in individually ventilated cages (transparent and with a top filter isolator) with a bed of sterilized wood bedding and were allowed access to water and chow *ad libitum*. The mice used in the experiments were all 6- to 8-week-old (18 - 20 g) males. All experiments were conducted in a blinded manner, and the mice were randomly allocated to each group for further research. All the mice were killed by excessive carbon dioxide inhalation at the end of the experiments.

Mouse PHG was established using a portal vein ligation (PVL) technique as previously described 5. At least 5 mice were included in each group. The portal vein was carefully isolated, and calibrated constriction was performed using a single ligature of 3-0 silk around the portal vein and a 20-gauge blunt-tipped needle under anesthesia by ketamine and xylazine mixed anesthetic. The needle was then removed, leaving a calibrated stenosis of approximately 50% of its diameter in the initial portal vein. In the sham operation (SO, as the control group) group, the same operation was performed without ligation after the portal vein was isolated. After the operation, all the animals were housed in cages and allowed free access to food and water for 2 weeks. In some experiments, the mice were treated for 2 weeks after the operation with the following reagents or drugs according to the experimental needs: for NF-κB inhibition, the mice were intraperitoneally injected with Bay11708 (BAY, Calbiochem, La Jolla, CA, USA) at 200 μg/per mouse daily for 2 weeks; for TNF-α inhibition, the mice were intraperitoneally injected with infliximab (IFX, Janssen Biotech, Horsham, PA, USA) at 5 mg/kg per day for 2 weeks; for RIPK1 inhibition, the mice were intraperitoneally injected with 2 mg/kg necrostatin-1 (Nec-1, HY-15760, MedChemExpress, NJ, USA) per day for 2 weeks; for above experiments of the inhibitors, the mice in the control group (vehicle group) were intraperitoneally injected with an equal volume of phosphate-buffered saline (PBS) daily for 2 weeks; for Drp1 inhibition, the mice were intraperitoneally injected with 20 mg/kg Mdivi-1 (dissolved in DMSO, HY-15886, MedChemExpress), and the control animals (vehicle) were injected with an equal volume of DMSO; for ROS scavenging, the mice were intraperitoneally injected with Mito-TEMPO (MT, dissolved in PBS, 10 mg/kg, HY-112879, MedChemExpress) every other day for 2 weeks, and the mice in the control group (vehicle) were intraperitoneally injected with an equal volume of PBS; for NOX2 blockade, 50 mg/kg GSK2795039 (GSK, dissolved in 20% DMSO, 20% Tween 80 and 60% polyethylene glycol 200, HY-18950, MedChemExpress) was administered by intraperitoneal injection daily for 2 weeks; for pan-caspase inhibition, the pan-caspase inhibitor benzyloxycarbonyl-Val-Ala-Asp (OMe) fluoromethylketone (Z-VAD-FMK, dissolved in 10% DMSO, 40% PEG300, 5% Tween-80 and 45% saline, 10 mg/kg daily; HY-16658B; MedChemExpress) was administered by intraperitoneal injection for 2 weeks, and the mice in the control group (vehicle) were intraperitoneally injected with an equal volume of the abovementioned solvents daily for 2 weeks.

### Sample collection

The PHG mice and the control mice were anesthetized and euthanized. The entire stomach was carefully removed, rinsed thoroughly and opened on its lesser curvature longitudinally to expose the mucosa, and the gastric injury indices were analyzed 4, 5: 0, normal; 1, mucosa with erosion; 2, mucosa with ulcers (< 1 mm); 3, mucosa with ulcers (1-2 mm); 4, mucosa with ulcers (3-4 mm); and 5, mucosa with ulcers (> 5 mm). Subsequently, the mucosal layers were harvested using glass slides and stored at -80 °C for protein and mRNA analyses. For histological analysis, the entire stomach was fixed in 10% neutral buffered formalin to prepare paraffin sections. The gastric specimens obtained from healthy volunteers and PHG patients were stored at -80 °C (for protein and mRNA analyses) or were fixed in 10% neutral buffered formalin (for paraffin sections).

### Histopathological staining

For histological analysis, 3 μm sections were stained with hematoxylin and eosin (H&E). For immunohistochemical (IHC) staining, 3 μm thick sections were cut, placed on charged slides and then processed as previously described 2, 5. The slides were incubated overnight at 4 °C with the corresponding antibodies against FADD (14906-1-AP, Proteintech, Proteintech Group, IL, USA), NLRP3 (A5652, A12694, ABclonal, Wuhan, China), 4-hydroxynonenal (4-HNE, ab46545, ab48506, Abcam, Cambridge, MA, USA), NOX1 (ab131088, Abcam), NOX2 (A1636, ABclonal), NOX4 (14347-1-AP, Proteintech), 8-OHdG (sc-393871, Santa Cruz, CA, USA), RIPK3 (A5431, ABclonal), LDHA (19987-1-AP, Proteintech), and ASC/TMS1 (A16672, ABclonal). Immunofluorescence (IF) staining of the samples was performed using primary antibodies against FADD (14906-1-AP, Proteintech), CK18 (cytokeratin 18, GTX105624, GeneTex, Alton Pkwy Irvine, CA, USA), caspase-8 (AF6442, Affinity, Cincinnati, OH, USA; 66093-1-Ig, Proteintech), TOMM20 (ab186735, ab283317, Abcam), RIPK1 (A7414, ABclonal), Drp1 (ab156951, Abcam; 8570S, Cell Signaling Technology, Danvers, MA, USA), Fis1 (A5821, ABclonal), p-Drp1^S616^ (AP0849, ABclonal), NOX2 (A1636, ABclonal), IL-1β (ab283818, Abcam), and NLRP3 (A5652, A12694, ABclonal). The targeted proteins were assessed using a related secondary antibody (Alexa Fluor 488-conjugated goat anti-rabbit, A-11008; Alexa Fluor 488-conjugated goat anti-mouse, A-11001; Alexa Fluor 594-conjugated goat anti-rabbit, A-11012; Alexa Fluor 594-conjugated goat anti-mouse, A-11005) as previously described 32, and the nuclei were counterstained with 2 mg/ml 4',6-diamidino-2-phenylindole dihydrochloride (DAPI, Molecular Probes, Eugene, OR, USA). For double IF staining, after the initial protein detection, the secondary target protein was detected on the same slides. Micrographs of the stained sections were captured by light microscopy and assessed utilizing ImageJ software (NIH, Bethesda, MD, USA).

For transmission electron microscopy (TEM), gastric tissues or cell samples were prepared and observed with a transmission electron microscope (Hitachi, H-800, Tokyo, Japan). The TEM images were acquired and stored digitally from a randomly selected pool of 6 fields. ImageJ software was used to measure the mitochondrial length/width ratio and average area. Briefly, 10 mitochondria in each image of a similar group were randomly selected, the mitochondrial length and width were measured based on the length parameter, and the mitochondrial area was measured with the area parameter.

### Primary cell isolation and cell culture

The entire mouse stomach was carefully removed and rinsed, the pylorus was ligated, and the primary gastric epithelial cells were then isolated using collagenase perfusion as previously described 5. In addition, an immortalized human gastric epithelial cell line named GES-1 was adopted as previously described 5. Isolated primary epithelial cells and GES-1 cells were cultured in RPMI 1640 medium supplemented with 10% fetal bovine serum, 100 units/ml penicillin and 100 μg/ml streptomycin in a humidified incubator at 37 °C with 5% CO_2_.

### Cell experiments

GES-1 cells and primary epithelial cells were cultured as described above. For shRNA treatment, cells were transfected with* FADD* shRNA lentiviral particles (m: sc-35351-V, h: sc-35352-V; Santa Cruz) according to the manufacturer's instructions. After 24 h of incubation, the transfection medium was replaced with regular culture medium. For some cell experiments, GES-1 cells were treated with TNF-α (80 ng/ml; Sigma, St. Louis, MO, USA) for 8 h according to the experimental needs. For Drp1 inhibition and NLRP3 inhibition respectively, 10 μM Mdivi-1 (a Drp1 inhibitor, HY-15886, MedChemExpress) or 10 μM MCC950 (a NLRP3 inhibitor, HY-12815, MedChemExpress) were added prior to TNF-α administration. For flow cytometric experiments, primary epithelial cells from the indicated groups were isolated and transfected with *FADD* shRNA lentiviral particles. These cells were then stained with the commercially available anti-NLRP3 antibody, and flow cytometric analysis was performed with a BD FACSCalibur and BD FACSAria (Becton Dickinson, NJ, USA) according to the manufacturer's instructions. The data were analyzed using FCS Express FlowJo 7.6 software.

### Confocal microscopy

For Mito-Tracker staining, cells were cultured on confocal culture dishes overnight, stained with 400 nM Mito-Tracker Red CM-H2XRos or Mito-Tracker Green FM (40740ES50, 40742ES50, Yeasen) for 30 min at 37 °C, and then washed three times with warm buffer. For MitoSox and Mito-Tracker double staining, cells were stained with Mito-Tracker Green FM and MitoSox for 20 min at 37 °C, and nuclei were then counterstained with Hoechst. The analyses were conducted using a Zeiss LSM880/800 confocal microscope. Mitochondrial fragmentation and morphology were assessed using ImageJ software, and the results were averaged for 10 cells per sample. For double IF staining, the cells were then fixed with 4% paraformaldehyde for 20 min and permeabilized with 0.25% Triton X-100 for 15 min. After the cells were blocked with 5% BSA (Solarbio), they were incubated with the appropriate primary antibodies overnight at 4 °C in PBS containing 1% BSA. After three washes in PBS with Tween 20 (PBST), the cells were incubated with secondary antibodies in 1% BSA for 2 h at 37 °C and rinsed in PBST. Nuclei were then counterstained with DAPI, and analyses were conducted using a Zeiss LSM880/800 confocal microscope.

### Western blotting

Total mucosal or cells proteins were analyzed by western blotting using the following antibodies: anti-FADD (14906-1-AP, Proteintech), anti-caspase-8 (AF6442, Affinity; 66093-1-Ig, Proteintech), anti-RIPK1 (A7414, ABclonal), anti-RIPK3 (A5431, ABclonal), anti-Drp1 (ab156951, Abcam; 8570S, Cell Signaling Technology), anti-Fis1 (A5821, ABclonal), anti-Mff (17090-1-AP, Proteintech), anti-Mfn1 (13798-1-AP, Proteintech), anti-TOMM20 (ab186735, ab283317, Abcam), anti-Mfn2 (A12771, ABclonal), anti-OPA1 (A9833, ABclonal), anti-p-Drp1^S616^ (AP0849, ABclonal; AF8470, Affinity), anti-p-Drp1^S637^ (AP0812, ABclonal; DF2980, Affinity), anti-Cox IV (A6564, ABclonal), anti-NLRP3 (A5652, A12694, ABclonal), anti-caspase-1 (A0964, ABclonal; ab207802, Abcam), anti-4-HNE (ab46545, ab48506, Abcam), anti-p-TAK1 (9339, Cell Signaling Technology), anti-TAK1 (5206, Cell Signaling Technology), anti-p-NF-κBp65 (3033s, Cell Signaling Technology), anti-NF-κBp65 (8242, Cell Signaling Technology), anti-Glut1 (12939S, Cell Signaling Technology), anti-HK2 (ab209847, Abcam), anti-PKM2 (A20991, ABclonal), anti-LDHA (19987-1-AP, Proteintech), anti-PDK1 (18262-1-AP, Proteintech), anti-GSDMD (ab219800, Abcam; AF4012, Affinity), anti-NOX2 (A1636, ABclonal), anti-Mid51 (20164-1-AP, Proteintech), anti-Mid49 (28718-1-AP, Proteintech), anti-ASC/TMS1 (A16672, ABclonal), anti-NDUFA9 (20312-1-AP, Proteintech), anti-SDHA (14865-1-AP, Proteintech), anti-Cyt b (cytochrome b, 55090-1-AP, Proteintech), anti-COX I (ab133319, Abcam), anti-ATP5A (ab176569, Abcam) and anti-β-actin (sc-47778, Santa Cruz) antibodies. Horseradish peroxidase-conjugated secondary antibodies were used to detect the primary antibodies/antigen complexes. The signal was detected using enhanced chemiluminescence (ECL) western blotting detection reagents. After signal quantification using NIH ImageJ software, the results are expressed as a ratio to the loading control in densitometric units.

### Immunoprecipitation

Coimmunoprecipitation was performed using a Thermo Scientific Pierce Coimmunoprecipitation Kit (Thermo Scientific, Rockford, AL, USA) as previously described 32, 33. The following antibodies were used: anti-FADD (14906-1-AP; Proteintech), anti-caspase-8 (AF6442; Affinity; sc-81656; Santa Cruz), anti-RIPK1 (A7414; ABclonal), anti-Drp1 (ab156951; Abcam; 8570S; Cell Signaling Technology), anti-Fis1 (A5821; ABclonal) and anti-TOMM20 (ab186735, ab283317, ab78547; Abcam). In addition, normal IgG was added, and the samples were incubated at 4 °C overnight on a rotator. Then, 25 µl of G/A agarose beads was added to 200 µl of lysate, and the mixture was incubated for 3 h with gentle agitation at room temperature according to the manufacturer's protocol. The beads were rinsed, boiled and then removed by centrifugation at 14,000 × g for 12 min, after which the final samples were analyzed via western blotting.

### Ubiquitination assays

For the analysis of endogenous RIPK1 ubiquitination, a coimmunoprecipitation assay was performed as described above with an anti-RIPK1 antibody, and ubiquitination was detected by western blotting analysis with an anti-ubiquitin antibody as previously described 33.

### Seahorse assay

Changes in mitochondrial pressure and the glycolytic rate in cells under different conditions were determined using a Seahorse XF96 extracellular flux analyzer (Agilent, Wilmington, DE, USA). We measured mitochondrial respiration (for the oxygen consumption rate [OCR]) and the glycolytic rate (for the extracellular acidification rate [ECAR]) by using the Seahorse XF Cell Mito Stress Test Kit (103015-100, Agilent) and the Seahorse XF Glycolytic Rate Assay Test Kit (103710-100, Agilent), respectively. For mitochondrial respiration detection, the OCR of basal respiration represents the energy demand of the cell in the basal state, which represents the ATP synthesis capacity of the mitochondria to satisfy the energy demand of the cell. After the baseline OCR was measured, 1.5 μmol/L oligomycin, 1 μmol/L carbonyl cyanide-4 (trifluoromethoxy) phenylhydrazone (FCCP), 0.5 μmol/L rotenone and antimycin A (Rot/AA) were added to measure relevant parameters related to mitochondrial function, such as basal respiration, proton leakage, maximal respiration and nonmitochondrial oxygen consumption. For the glycolytic rate test, after the baseline extracellular acidification rate (ECAR) was measured, 0.5 μmol/L Rot/AA and 50 mmol/L 2-deoxyglucose (2-DG) were added sequentially. The glycolytic levels of the cells were determined by the glycoPER value. After treatment, DMEM culture medium (pH 7.4) (103575-100, Agilent) were preheated in a 37 °C water bath. The culture medium (pH 7.4) contained 10 mmol/L glucose, 1 mmol/L pyruvate and 2 mmol/L glutamine, and the cells were subsequently incubated for 1 h in 37 °C incubator without CO_2_ and finally tested on a Seahorse XF96 extracellular flux analyzer.

### ATP measurement

The ATP concentration in the gastric sections was measured using a bioluminescence assay kit (Beyotime Biotechnology, Shanghai, China) according to the manufacturer's protocol. In brief, gastric tissues were lysed in the appropriate lysis buffer. The supernatant was collected by centrifugation at 12,000 × rpm for 5 min at 4 °C. The ATP concentration in the tissues was examined by mixing 20 μl of the supernatant with 100 μl of luciferase reagent (luciferase can catalyze the generation of luminescence from ATP and luciferin) provided with the assay kit. The luminescence of each sample was measured on a microplate luminometer, and the measured ATP concentration is presented as nmol/mg of protein based on a standard curve of ATP.

### Luciferase reporter gene assay

A luciferase reporter gene assay was performed as previously reported 32. GES-1 cells were cotransfected with control shRNA (vector) or *FADD* shRNA lentiviral particles and pGL4.1 vectors containing the *NOX2* reporter followed by TNF-α or saline (as vehicle) treatment, and a Dual-Glo Luciferase Assay (Promega, Madison, WI, USA) was subsequently performed according to the manufacturer's instructions. Similarly, GES-1 cells were transfected with the *pcDNA3.1-p65-*vector or *pcDNA3.1-*vector (vector), and *NOX2* reporter luciferase analysis was subsequently performed.

### Energy metabolic analysis

Isolated primary gastric epithelial cells were washed three times with PBS, stored on dry ice and transported. Energy metabolism was detected and analyzed using an Acquity-I Xevo TQ-S instrument (Waters Corp., Milford, MA, USA) following the manufacturer's instructions based on the Technical Platform for Targeted Metabolism (UPLC-MS/MS, Metabo-Profile Biotechnology, Shanghai, China). Data analysis was further performed using MassLynx software (V4.1, Waters Corp.) and iMAP software (V1.0, Metabo-Profile Biotechnology).

### Microarray analysis

The microarray experiment was performed as previously described 32, 33. Total RNA from each sample was dissociated with TRIzol reagent (Invitrogen) according to the instructions and purified using a mirVana miRNA Isolation Kit (Ambion, Austin, TX, USA). cDNA labeled with Cy3dCTP was generated using the Eberwine linear RNA amplification method and an enzymatic reaction, and the double-stranded cDNA (dsDNA) products were subsequently purified and eluted with the PCR NucleoSpin Extract II Kit according to the instructions. The eluted dsDNA was then vacuum evaporated to 16 ml and subjected to 40 ml of transcription reaction with the T7 Enzyme Mix at 37 °C for 14 hours according to the instructions. Klenow enzyme labeling tactics were used, and the samples were subsequently subjected to reverse transcription using CbcScript II reverse transcriptase. Finally, array hybridization was performed in an Agilent hybridization oven overnight. Subsequently, the GeneSpring software V12 array (Agilent) was used to summarize and normalize the data. To select the differentially expressed genes, we used threshold values of ≥ 1.5 fold change and ≤-1.5 fold change and a Benjamini-Hochberg-corrected *P* value of 0.05. The Adjust Data function of CLUSTER 3.0 software was used for log2 transformation and hierarchical clustering of the data, and Java TreeView (Stanford University School of Medicine, Stanford, CA, USA) was used for tree visualization.

### Separation of the cytoplasm and mitochondria

Mitochondria were isolated from gastric mucosal epithelial cells or GES-1 cells using a Cell Mitochondria Isolation Kit (ab110170, Abcam). In brief, the cells were plated in a 6-well plate, exposed to different concentrations of the cytotoxic compounds for the indicated times, washed three times with precooled PBS and lysed with Cell Mitochondria Isolation buffer on ice following the standard protocol. Parts of the mitochondria and cytoplasm were then separated by centrifugation at 600 × g for 15 min at 4 °C. Subsequently, the supernatant was further ground and centrifuged at 11,000 × g for 10 min at 4 °C. The pellet (the mitochondrial fraction) was collected and resuspended in mitochondrial lysis buffer for further analysis. The remaining supernatant was then centrifuged at 12,000 × g for 15 min at 4 °C as the cytosolic fraction for subsequent research.

### Determination of oxidative stress

The intracellular ROS levels were measured with a Reactive Oxygen Species Assay Kit (Beyotime Biotechnology). In brief, the treated cells were incubated with 2,7-dichlorodihydrofluorescein diacetate (DCFH-DA, diluted 1: 1000 in RPMI 1640 medium) for 20 min at 37 °C and then analyzed using a fluorescence spectrophotometer at 488 nm excitation and 525 nm emission wavelengths.

The levels of MDA were measured using a malondialdehyde (MDA) assay kit (ab118970, Abcam), and the MDA concentrations were measured using a spectrophotometer (BioTek-Epoch2, USA) at 532 nm.

A 5 mM MitoSOX™ (Red Mitochondrial Superoxide Indicator) reagent stock solution (Thermo Fisher) was diluted in RPMI 1640 medium to obtain a 5 μM MitoSOX™ reagent working solution, and the cells were then incubated with 1.0 ml of this MitoSOX™ reagent working solution for 10 min at 37 °C in the dark. The cells were then gently washed three times with PBS, and the nuclei were counterstained with Hoechst 33258 (10 μg/ml, Beyotime Biotechnology). Images were acquired with a fluorescence microscope (Imager Z2, Zeiss, Germany).

The mitochondrial membrane potential (ΔΨm) was determined using a mitochondrial membrane potential JC-1 fluorescence assay kit (Beyotime Biotechnology). The cells were incubated at 37 °C for 20 min with a JC-1 kit, washed twice with cold JC-1 staining buffer and placed in fresh medium. In healthy cells with a high ΔΨm, JC-1 forms complexes known as JC-1 aggregates, whereas in cells with a low ΔΨm, JC-1 remains monomeric. Images were viewed and analyzed at 490 nm excitation and 530 nm emission for green (JC-1 monomers) and at 540 nm excitation and 590 nm emission for red (JC-1 aggregates).

For the measurement of oxidized mitochondrial DNA (ox-mtDNA), mtDNA was first purified from the mitochondrial fraction of primary epithelial cells using an AllPrep DNA/RNA Mini Kit (Qiagen, Germany). The 8-OHdG content was then quantified using an 8-OHdG quantification kit (Cell Biolabs, San Diego, CA, USA) following the manufacturer's instructions.

For antioxidant enzyme activity analysis, the activity of SOD (superoxide dismutase, Beyotime, S0101S) and the GSH/GSSG ratio (GSH/GSSG ratio detection assay kit, ab205811, Abcam) were measured by using the appropriate assay kits.

### Ca^2+^ concentration detection and enzyme-linked immunosorbent assay (ELISA)

The intracellular Ca^2+^ concentration ([Ca^2+^]i) was quantified by incubating the cells with 5 μM Fluo-3 AM (ab145254, Abcam) for 40 min at room temperature and two washes with PBS. The fluorescence signal was detected with a Leica STELLARIS STED confocal microscope (Fluo-3 AM was excited alternately at 506 nm, and emission was monitored at 526 nm); [Ca^2+^]i was calculated as the ratio of F506 to F526. The mitochondrial Ca^2+^ concentration ([Ca^2+^]m) was recorded using Rhod-2 AM (ab142780, Abcam). The cells were incubated with 5 μM Rhod-2 AM for 40 min at room temperature and detected by confocal microscopy (Rhod-2 AM was excited at 552 nm, and emission was monitored at 581 nm); [Ca^2+^]m is expressed as the ratio of F552 to F581. The IL-1β and IL-18 concentrations were measured using ELISA kits (R&D Systems, Minneapolis, MN, USA) following the manufacturers' instructions.

### Statistical analysis

All the experiments were repeated at least three times with similar results, and different representative specimens were adopted and presented for the related images or bands in the current experiments. The results are expressed as the means ± standard errors of the means (SEMs). With GraphPad Prism software, statistical analyses were performed by Student's two-tailed paired *t* test, one-way ANOVA (more than two groups of data, single factor) or two-way ANOVA (more than two groups of data, two factors) with repeated measures followed by Bonferroni's comparison *post hoc* test, and post hoc tests were run only if *F* achieved *P* < 0.05. A value of *P* < 0.05 was considered to indicate statistical significance.

## Results

### FADD combines with caspase-8 to induce gastric mucosal injury in PHG

**Figure 1:**
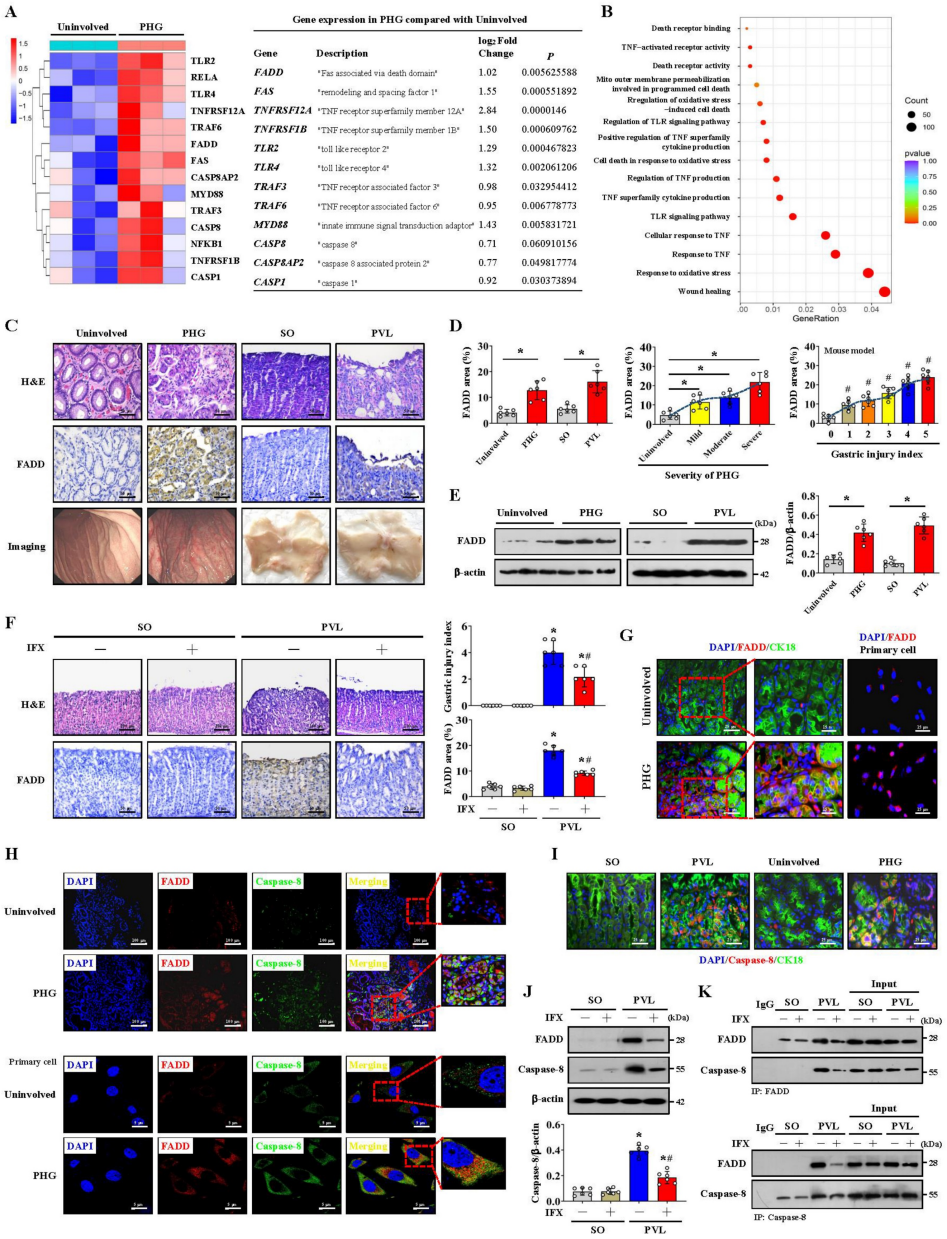
** FADD combines with caspase-8 to induce gastric mucosal injury in PHG.** (A) Two-dimensional hierarchical clustering results of the genes of death receptor family members between portal hypertensive gastropathy patients (as PHG) and healthy volunteers (as Uninvolved) (n = 3 per group). The fold changes and *P* values of the indicated mRNAs in PHG tissues relative to those in normal tissues were also presented. (B) Enrichment of signaling pathways according to the RNA sequencing assay. The wound healing pathway, TNF-α-related signaling pathway and oxidative stress-related network were strongly enriched. (C) Representative images of H&E (hematoxylin and eosin) staining, Fas-associated protein with death domain (FADD) immunohistochemistry (IHC) staining and gastric imaging of the normal gastric mucosal tissue and gastropathic mucosal tissue in the indicated sections were presented (n = 6 per group). (D) The percentage of FADD area was calculated in PHG patients compared to healthy controls and in mice with PVL compared to control (SO, sham operation) mice based on the IHC staining shown in (C). The FADD levels were positively correlated with the degree or severity of gastric mucosal injury in PHG patients and mice with PVL. n = 6 per group; *P < 0.05; #*P* < 0.05 versus the group with a gastric injury index of 0. (E) The FADD levels in the indicated gastric mucosa were determined by western blotting in three pairs of representative specimens. The ratio of densitometric units of the normalized FADD/β-actin was also analyzed. n = 6 per group, *P < 0.05. (F) Representative photographs of H&E staining and FADD IHC staining of gastric mucosal sections from SO and mice with PVL treated with or without the TNF-α inhibitor infliximab (IFX). The histogram quantifies the gastric injury index and the FADD area. n = 6 in each group. *P < 0.05 versus SO mice; #*P* < 0.05 versus mice with PVL without IFX treatment. (G) Double immunofluorescence (IF) staining of cytokeratin 18 (CK18, green) and FADD (red) indicated that FADD was located mainly in the epithelial cells of PHG patients. FADD (red) IF staining was also performed on primary isolated epithelial cells from the PHG and uninvolved tissues. Nuclei (blue) were counterstained with 4',6-diamidino-2-phenylindole dihydrochloride (DAPI). (H) Double IF staining of FADD (red) and caspase-8 (green) in the indicated groups revealed that they were located in similar cells from PHG patients. Nuclei (blue) were counterstained with DAPI. (I) Costaining of cytokeratin 18 (CK18, green) and caspase-8 (red) in human samples and mouse models suggested that caspase-8 was highly expressed in gastric epithelial cells from PHG patients and mice with PVL. Nuclei (blue) were counterstained with DAPI. (J) Western blotting analysis demonstrated that IFX suppressed the expression of FADD and caspase-8 in mice with PVL. The ratio of densitometric units of the normalized caspase-8/β-actin was also analyzed. n = 6 per group; *P < 0.05 versus SO mice; #*P* < 0.05 versus mice with PVL without IFX treatment. (K) Primary epithelial lysates from SO mice and mice with PVL were immunoprecipitated with an anti-FADD antibody and then analyzed with a caspase-8 antibody (upper panel). Primary epithelial lysates from the indicated mice were immunoprecipitated with an anti-caspase-8 antibody and then analyzed with a FADD antibody (lower panel). n = 6 per group.

We examined the transcriptomes of gastric mucosal biopsy tissue isolated from volunteers and PHG patients and found that the gastric mucosal samples from the PHG group had higher levels of TNF-α-related elements and signal transducers (e.g., FADD and caspase-8) than did the normal samples (Uninvolved) (**Figure 1A**). Moreover, the wound healing pathway, TNF-α-related signaling pathways and oxidative stress-related network were markedly enriched (**Figure 1B**). A loss of the preserved architecture of the gastric mucosa and mucosal injury associated with upregulated FADD expression were found in the PHG patients (**Figure 1C-1E**). A PHG mouse model established by partial portal vein ligation (PVL) was used, and we also verified that the gastric mucosa showed architecture loss and injury and that the FADD expression was upregulated in the mice with PHG compared with the control mice from the SO group (sham operation) (**Figure 1C-1E**).

Moreover, the FADD level was positively correlated with the severity of gastric mucosal injury in PHG patients and mice with PVL (**Figure 1D**). Based on the involvement of TNF-α-related signaling pathways, the selective TNF-α inhibitor infliximab (IFX) was used. Histopathological analysis revealed that the gastric injury index and FADD expression were decreased in the mice with PVL after IFX administration (**Figure 1F**). Costaining confirmed that enhanced FADD was located mainly in gastric epithelial cells in PHG (**Figure 1G, Figure S1A**). Double staining further revealed that, in association with FADD upregulation, caspase-8 was also highly expressed in similar gastric epithelial cells from PHG patients and mice with PVL (**Figure 1H, 1I, Figure S1A**). Western blotting analysis demonstrated that the levels of FADD and caspase-8 were decreased in the mice with PVL after IFX administration (**Figure 1J**), and immunohistochemical analysis showed that IFX repressed the expression of caspase-8 in the mice with PVL (**Figure S1B**). Moreover, direct enhancement of the interaction between FADD and caspase-8 was detected by immunoprecipitation in the mice with PVL but not in the SO mice, while IFX administration attenuated this interaction in the mice with PVL (**Figure 1K**). These results show that FADD combines with caspase-8 to contribute to gastric mucosal injury in PHG.

### FADD/caspase-8 contributes to gastric epithelial mitochondrial dysfunction and oxidative stress

**Figure 2:**
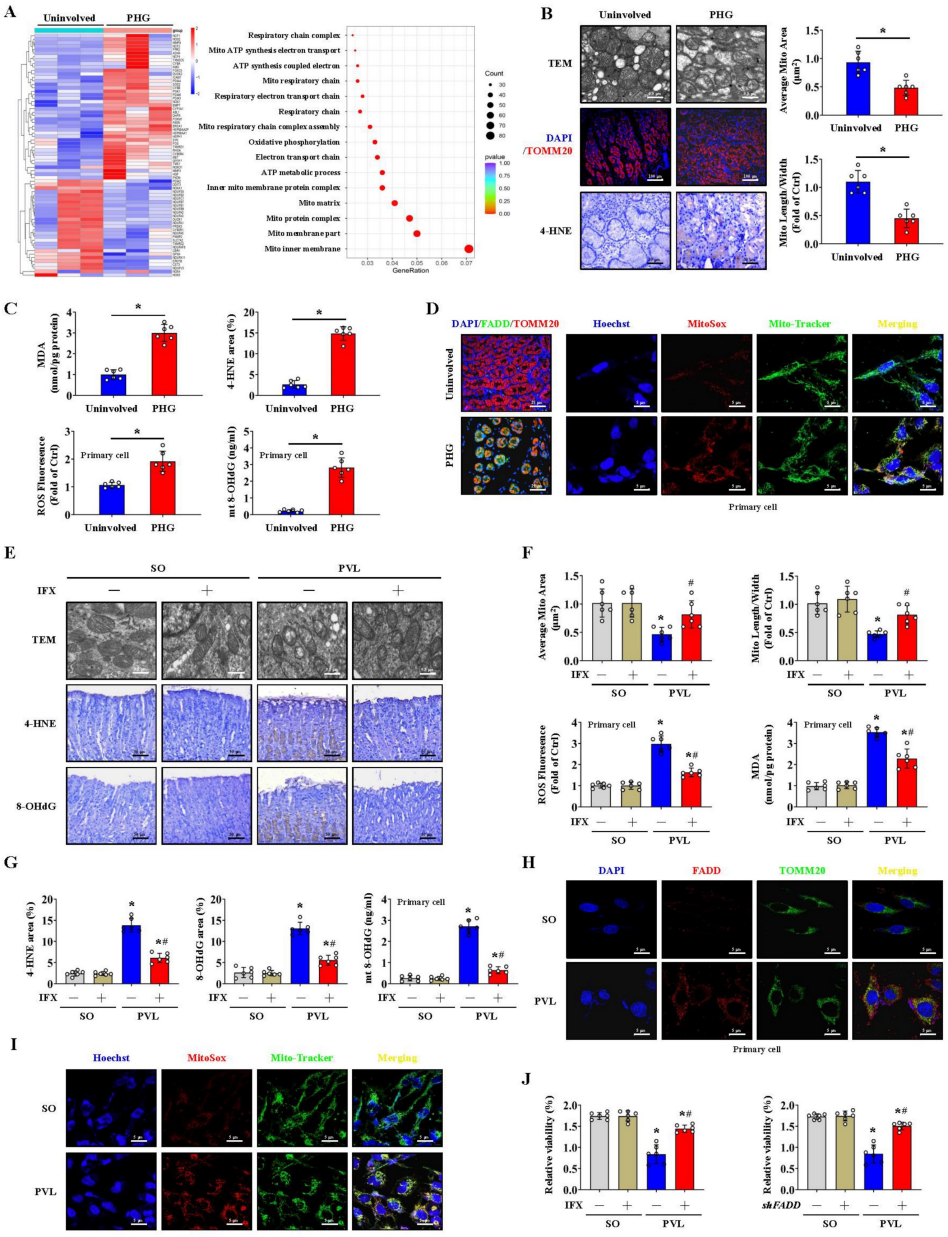
** FADD/caspase-8 contributes to gastric epithelial mitochondrial dysfunction and oxidative stress.** (A) Heatmap of the differentially expressed genes in the PHG patients and healthy volunteers (as Uninvolved). Enrichment of signaling pathways according to the RNA sequencing assay showed that multiple mitochondrial structure- and respiration-related pathways were enhanced in the gastric mucosa of PHG (n = 3 per group). (B) Representative images of TEM images of mitochondria, IF staining for TOMM20 (red), and IHC staining for 4-HNE (brown) in the gastric mucosa of PHG patients and healthy volunteers (as Uninvolved). The histograms showed the quantification of the average mitochondrial area and the mitochondrial length/width ratio in the indicated groups. n = 6 per group, *P < 0.05. (C) The MDA levels and 4-hydroxynonenal (4-HNE) area (from IHC staining) revealed enhanced oxidative stress in PHG patients (upper panel). The levels of ROS and mitochondrial 8-OHdG (mt 8-OHdG) in primary epithelial cells isolated from human sections were shown (lower panel). n = 6 per group, *P < 0.05. (D) Double IF staining of FADD (green) and TOMM20 (red) suggested that FADD was located in mitochondria in the gastric mucosa of PHG patients, nuclei (blue) were counterstained with DAPI. Costaining of MitoSox (red) and Mito-Tracker (green) suggested that ROS signaling was located in mitochondria in the primary epithelial cells of PHG patients, nuclei (blue) were counterstained with Hoechst. (E) Representative TEM images of mitochondria and IHC staining for 4-HNE and 8-OHdG (brown) in mouse models verified that IFX attenuated the degree of mitochondrial damage and mitochondrial oxidative stress. (F) Quantitative analysis of the average mitochondrial area and mitochondrial length/width index determined via TEM; the ROS and MDA levels in the corresponding primary epithelial cells were also presented. n = 6 in each group; *P < 0.05 versus SO mice; #*P* < 0.05 versus mice with PVL without IFX treatment. (G) The 4-HNE and 8-OHdG areas from (E) and the mt 8-OHdG level from the indicated primary epithelial cells revealed that IFX alleviated mitochondrial oxidative stress in mice with PVL. n = 6 in each group; *P < 0.05 versus SO mice; #*P* < 0.05 versus mice with PVL without IFX treatment. (H) Double IF staining of FADD (red) and TOMM20 (green) in primary epithelial cells from mouse models showed that FADD was located in the mitochondria of mice with PVL. (I) Costaining of MitoSox (red) and Mito-Tracker (green) revealed that ROS were generated from mitochondria in primary epithelial cells. Nuclei (blue) were counterstained with Hoechst. (J) Analysis of epithelial cell viability in the indicated groups via a CCK-8 assay showed that epithelial cell viability was markedly decreased in mice with PVL, whereas IFX treatment or *FADD* knockdown via *shFADD* restored epithelial cell viability. n = 6 in each group, *P < 0.05 versus SO mice; #*P* < 0.05 versus mice with PVL without IFX treatment or *shFADD* transfection.

A heatmap and enrichment analysis showed that multiple mitochondrial structure- and respiration-related pathways were enhanced in the gastric mucosal samples of PHG patients (**Figure 2A**). TEM revealed mitochondrial swelling, fragmented morphology and disruption of membrane integrity with broken or absent cristae in the epithelial cells of PHG patients and mice with PVL (**Figure 2B, 2E**). Moreover, 4-HNE, MDA, and intracellular ROS levels and mitochondrial 8-OHdG (mt 8-OHdG) were significantly enhanced in the gastric mucosa or epithelial cells of PHG patients (**Figure 2B, 2C**), and FADD was found to be located mainly in mitochondria in the mucosa of the PHG patients and mice with PVL (**Figure 2D, 2H**). MitoSox analysis revealed that mtROS levels were increased in the gastric mucosal samples of PHG patients, and Mito-Tracker staining revealed an increase in mitochondrial fragmentation in PHG patients (**Figure S2A**). Costaining of MitoSox and Mito-Tracker revealed that the increase in ROS generation was generated from the mitochondria in the primary epithelial cells of PHG patients (**Figure 2D**). Obvious abnormal or damaged mitochondria and enhanced oxidative stress (detected by 4-HNE, 8-OHdG, ROS, MDA and mt 8-OHdG levels) were observed in the epithelial cells from the mice with PVL, while IFX administration alleviated these symptoms (**Figure 2E-2G**). Double staining of MitoSox and Mito-Tracker also revealed that ROS were generated from mitochondria in the primary epithelial cells of mice with PVL (**Figure 2I, Figure S2B**), while *FADD* knockdown by *shFADD* blocked this process (**Figure S2B**). Moreover, an increase in mitochondrial fragmentation was detected in the epithelial cells of mice with PVL, which was associated with increased mtROS levels, and *shFADD* decreased the extent of mitochondrial fragmentation and mtROS levels in the epithelial cells of mice with PVL (**Figure S2C, S2D**). Analysis of epithelial cell viability via a CCK-8 assay showed that epithelial cell viability was markedly decreased in the mice with PVL, while IFX treatment or *shFADD* transfection restored epithelial cell viability (**Figure 2J**). These findings show that FADD/caspase-8 translocates to mitochondria to mediate gastric epithelial mitochondrial dysfunction and oxidative stress in PHG.

### The formation of the FADDosome mediates mitochondrial dysfunction via Drp1-dependent mitochondrial fission in PHG

**Figure 3:**
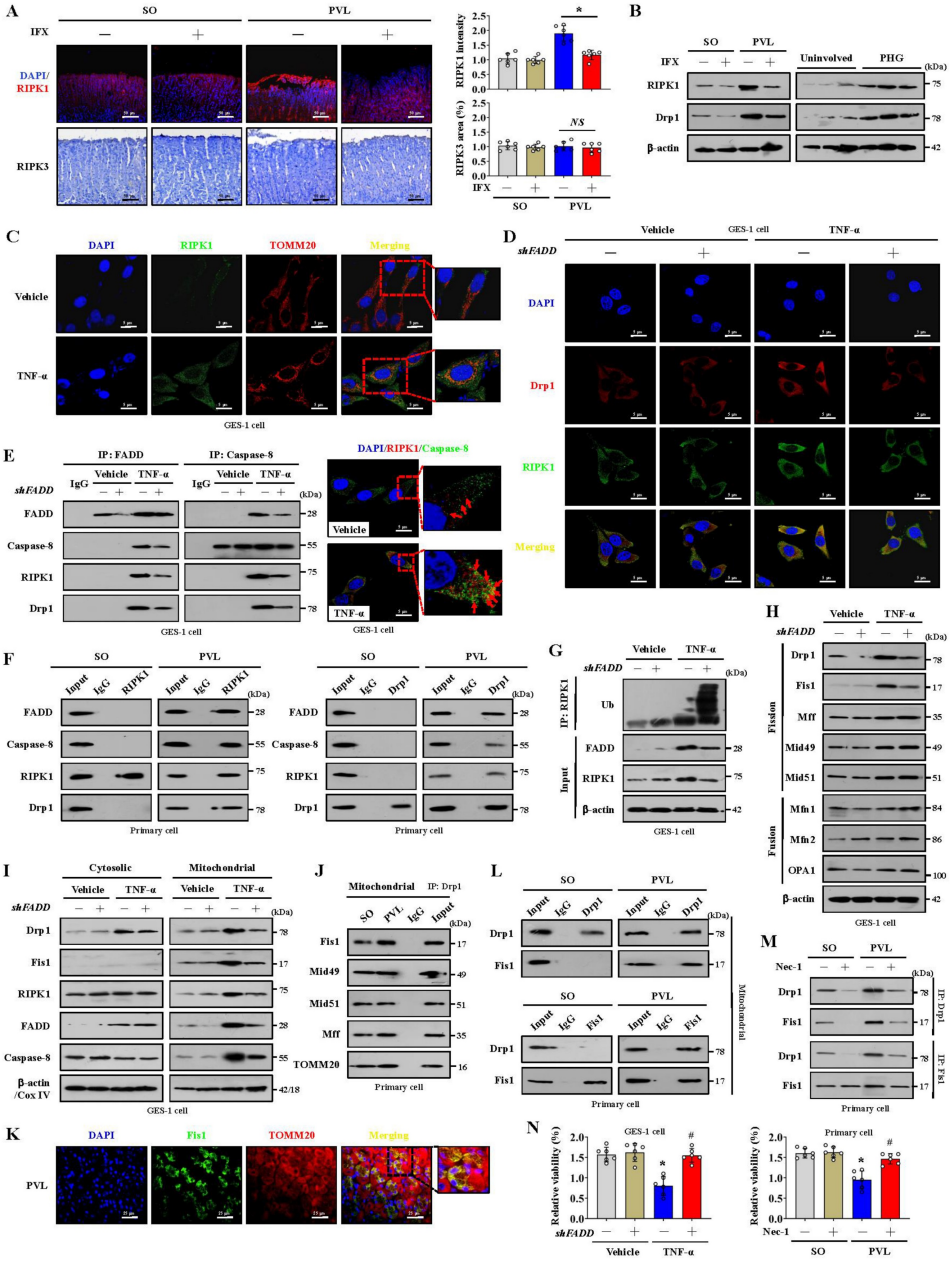
** The formation of the FADDosome mediates mitochondrial dysfunction via Drp1-dependent mitochondrial fission in PHG.** (A) Representative photographs of RIPK1 IF staining and RIPK3 IHC staining in mouse models following IFX treatment and quantitative analysis of RIPK1 and RIPK3 expression. n = 6 per group, *P < 0.05; *NS*, not significant. (B) Western blotting for RIPK1 and Drp1 in mouse models (left) and human sections (right) revealed that increased RIPK1 and Drp1 expression was found in mice with PVL and PHG patients, and IFX treatment repressed that upregulation in mice with PVL. n = 6 per group. (C) Double IF staining of RIPK1 (green) and TOMM20 (red) in GES-1 cells suggested that TNF-α promoted the upregulation of RIPK1 in mitochondria. Nuclei (blue) were counterstained with DAPI. (D) Costaining of Drp1 (red) and RIPK1 (green) in GES-1 cells revealed that the upregulation of RIPK1 and its interaction with Drp1 induced by TNF-α were reversed by *shFADD*. Nuclei (blue) were counterstained with DAPI. (E) Interactions among FADD, caspase-8, RIPK1 and Drp1 in GES-1 cells were detected by IP (left panel), which revealed that the interaction induced by TNF-α was attenuated by *shFADD*. Costaining of RIPK1 (red) and caspase-8 (green) determined by confocal microscopy indicated (right panel) the mutual interaction between caspase-8 and RIPK1 via TNF-α. Nuclei (blue) were counterstained with DAPI. n = 6 per group. (F) Lysates of primary cells isolated from mouse models were subjected to IP with an anti-RIPK1 (left) or anti-Drp1 (right) antibody and analyzed by immunoblotting with the indicated antibodies. The results demonstrated that interactions among FADD, caspase-8, RIPK1 and Drp1 were enhanced in mice with PVL. n = 6 per group. (G) Immunoprecipitation of the indicated GES-1 cell lysates (transfected with vector or *shFADD* and treated or not treated with TNF-α) for RIPK1 ubiquitination analysis revealed that *shFADD* enhanced RIPK1 ubiquitination and decreased RIPK1 protein level in TNF-α-treated GES-1 cells. n = 6 per group. (H) Western blotting for mitochondrial fission regulator (Drp1, Fis1, Mff, Mid49, Mid51) and mitochondrial fusion regulator (Mfn1, Mfn2, OPA1) proteins in GES-1 cells suggested that *shFADD* treatment only affected the expression of Drp1 and Fis1 induced by TNF-α. n = 6 per group. (I) The levels of Drp1, Fis1, FADD, caspase-8 and RIPK1 were isolated from the mitochondrial and cytosolic fractions of GES-1 cells (transfected with vector or *shFADD* and treated with TNF-α or not), as detected by western blotting, which verified that *shFADD* decreased their expression induced by TNF-α in the mitochondrial lysates. n = 6 per group. (J) The interactions among mitochondrial fission components (Drp1, Fis1, Mff, Mid49 and Mid51) in isolated mitochondrial sections from mouse models determined by IP suggested that Drp1 could bind to fission elements to enhance mitochondrial fission. n = 6 per group. (K) Double IF staining of Fis1 (green) and TOMM20 (red) with DAPI (blue) counterstaining for nuclei in gastric sections from the PVL mouse model showing the location of Fis1 in mitochondria. (L) Isolated mitochondrial fractions of primary epithelial lysates from mouse models were immunoprecipitated with anti-Drp1 or anti-Fis1 antibodies and then detected by anti-Drp1 and anti-Fis1 antibodies, respectively (n = 6 per group), which indicated that the interaction between Drp1 and Fis1 from mitochondria was enhanced in mice with PVL. (M) IP analysis of the interaction between Drp1 and Fis1 in primary epithelial lysates from mouse models showed that necrostatin-1 (Nec-1, an inhibitor of RIPK1) treatment attenuated the binding of Drp1 and Fis1 in mice with PVL. n = 6 per group. (N) Cell viability from the indicated groups analyzed by CCK-8 assay showed the cell viability from mice with PVL or TNF-α-treated GES-1 cells was markedly repressed, and *FADD* knockdown by *shFADD* or Nec-1 reversed this effect. n = 6 in each group; *P < 0.05 versus the vehicle group or SO mice; #*P* < 0.05 versus TNF-α-treated cells without *shFADD* transfection or mice with PVL without Nec-1 treatment.

Mitochondrial dynamics might play an essential role in stress signaling 34. Increased RIPK1 and Drp1 expression rather than RIPK3 expression was found in the gastric mucosal samples of mice with PVL and PHG patients compared with that in the corresponding control groups, and the upregulation of RIPK1 and Drp1 in mice with PVL was reversed by IFX treatment (**Figure 3A, 3B**). Given the involvement of TNF-α-related signaling pathways in PHG, the inflammatory factor TNF-α was used, and we found that RIPK1 was upregulated and colocalized with TOMM20 and Drp1 in GES-1 cells following TNF-α treatment, whereas this upregulation of RIPK1 and its interaction with Drp1 could be reversed by *shFADD* (**Figure 3C, 3D**). The TNF-α-induced interactions among FADD, caspase-8, RIPK1 and Drp1 were attenuated following *shFADD* transfection, as shown by immunoprecipitation (IP), and double IF staining analysis further revealed mutual interactions between caspase-8 and RIPK1 following TNF-α treatment (**Figure 3E**). In PVL mouse models, we also confirmed the abovementioned mutual interaction by IP (**Figure 3F**), suggesting that FADD and caspase-8 recruit RIPK1 to form the FADDosome in the gastric epithelium of PHG. *ShFADD* enhanced RIPK1 ubiquitination and decreased RIPK1 protein level in TNF-α-treated GES-1 cells (**Figure 3G**), revealing that FADD enhances RIPK1 activity by inhibiting its ubiquitination. The elements related to mitochondrial dynamics were analyzed, and we showed that the mitochondrial fusion regulators mitofusin 1 (Mfn1), mitofusin 2 (Mfn2) and optic atrophy 1 (OPA1) were not affected by* shFADD*, although their expression was clearly increased by TNF-α. The mitochondrial fission elements Drp1 and Fis1 were affected by *shFADD* in TNF-α-treated cells (**Figure 3H**). By separating the cytoplasm and mitochondria, we found that FADD, caspase-8, RIPK1, Drp1 and Fis1 expression was increased in the mitochondrial lysates of TNF-α-treated cells, and these increases were blocked by *shFADD* (**Figure 3I**). Increased levels of Fis1 localized to the mitochondria in mice with PVL (**Figure 3K**), and the interaction between Drp1 and Fis1 from mitochondria was enhanced in the gastric epithelial cells from mice with PVL, although Drp1 could also bind to other fission elements, such as Mid49, Mid51 and Mff (**Figure 3J, 3L**). Necrostatin-1 (Nec-1), an inhibitor of RIPK1, downregulated Drp1 and Fis1 expression and attenuated the direct binding of Drp1 and Fis1 in mice with PVL (**Figure 3M**). Cell viability was clearly decreased in mice with PVL and TNF-α-treated GES-1 cells, while *FADD* knockdown via *shFADD* or Nec-1 restored the viability (**Figure 3N**). These results reveal that the formation of FADDosomes mediates mitochondrial dysfunction via Drp1-dependent mitochondrial fission.

### Blocking mitochondrial fission alleviates epithelial mitochondrial dysfunction and oxidative stress in PHG

**Figure 4:**
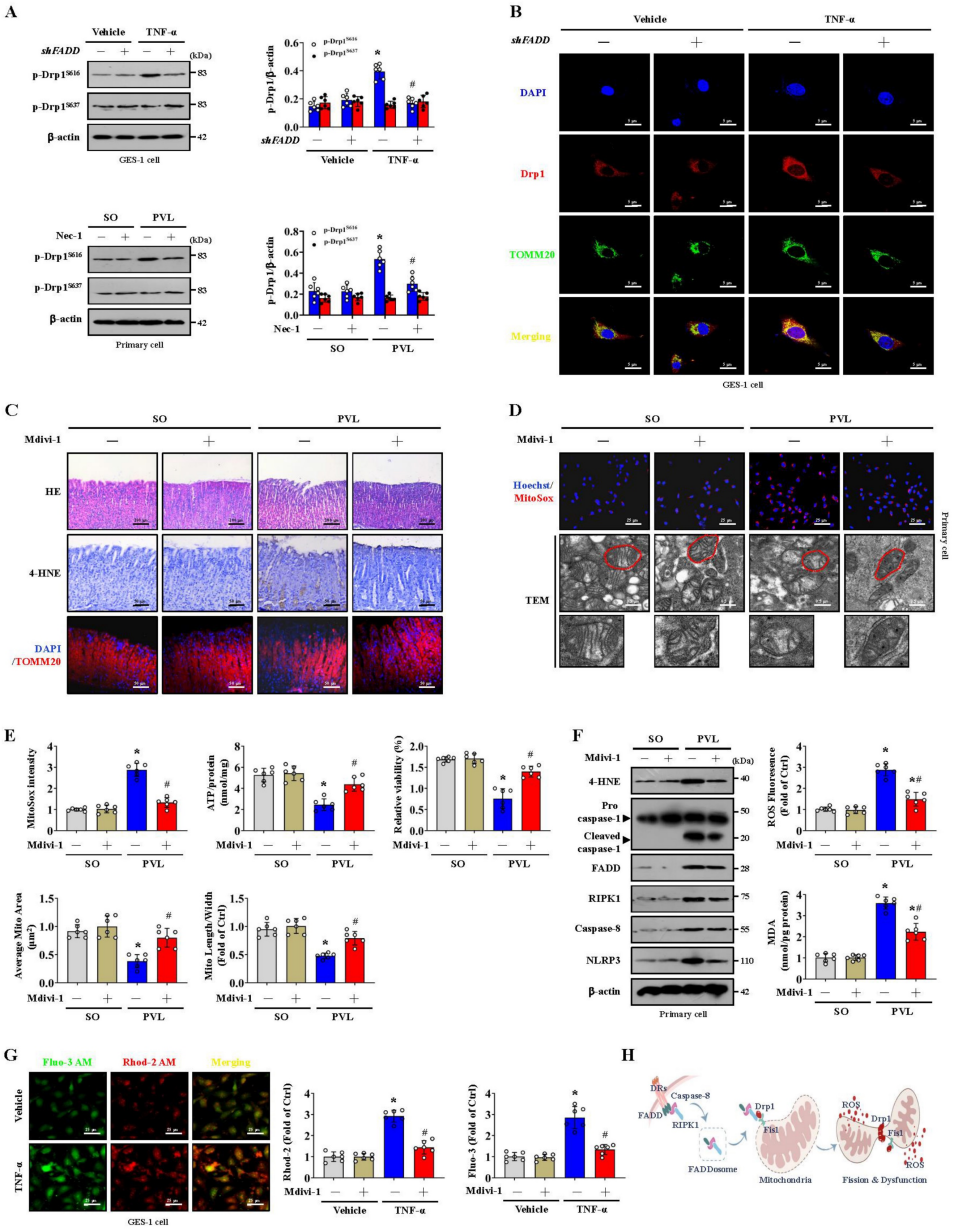
** Blocking mitochondrial fission alleviates epithelial mitochondrial dysfunction and oxidative stress in PHG.** (A) The levels of p-Drp1 (Ser616) and p-Drp1 (Ser637) in the indicated GES-1 cells and primary epithelial cells from mouse models detected by western blotting revealed that *FADD* knockdown by *shFADD* or Nec-1 reduced the phosphorylation of Drp1 at Ser616 induced by TNF-α treatment or PVL operation. The ratio of densitometric units of normalized p-Drp1/β-actin was determined. n = 6 per group; *P < 0.05 versus the vehicle group or SO mice; #*P* < 0.05 versus TNF-α-treated cells without *shFADD* or mice with PVL without Nec-1. (B) Confocal images revealed that *shFADD* decreased the TNF-α-induced mitochondrial translocation of Drp1, as indicated by localization of Drp1 (red) and TOMM20 (green) in GES-1 cells. Nuclei (blue) were counterstained with DAPI. (C) H&E staining, 4-HNE IHC staining (brown) and TOMM20 IF staining (red) of gastric mucosal tissues from mouse models indicated that the mitochondrial division inhibitor 1 (Mdivi-1) attenuated mucosal injury and 4-HNE expression in mice with PVL. n = 6 per group. (D) MitoSox staining (red, nuclei (blue) were counterstained with Hoechst) and mitochondrial ultrastructural features by TEM suggested that Mdivi-1 alleviated mtROS levels and restored abnormal or damaged mitochondria in the primary epithelial cells of mice with PVL. n = 6 per group. (E) The MitoSox intensity, ATP concentration and cell viability of primary isolated epithelial cells indicated that Mdivi-1 alleviated mtROS levels and increased ATP concentration and cell viability in mice with PVL (upper panel). The quantified mitochondrial area and the ratio of mitochondrial length to width were also detected (lower panel). n = 6 per group. *P < 0.05 versus SO mice; #*P* < 0.05 versus mice with PVL without Mdivi-1 treatment. (F) Representative western blotting for 4-HNE, procaspase-1, cleaved caspase-1, FADD, RIPK1, caspase-8 and NLRP3 showed that Mdivi-1 decreased the levels of 4-HNE, cleaved caspase-1 and NLRP3 without affecting the status of FADD, RIPK1 or caspase-8 in epithelial cells from mice with PVL. The MDA and ROS levels detection showed that Mdivi-1 attenuated mitochondrial oxidative stress in mice with PVL. n = 6 per group. *P < 0.05 versus SO mice; #*P* < 0.05 versus mice with PVL without Mdivi-1 treatment. (G) The cytosolic and mitochondrial Ca^2+^ levels in GES-1 cells treated with vehicle or TNF-α were measured with Fluo-3 AM (green) and Rhod-2 (red), respectively. Merged images (yellow) were also presented. The Ca^2+^ fluorescence intensity of GES-1 cells revealed that Mdivi-1 maintained Ca^2+^ homeostasis in TNF-α-treated cells. n = 6 per group. *P < 0.05 versus the vehicle group; #*P* < 0.05 versus TNF-α-treated cells without Mdivi-1 treatment. (H) Schematic diagram of FADDosome-regulated mitochondrial fission and dysfunction by Drp1/Fis1 signaling in PHG.

The phosphorylation of Serine 616 (Ser616) promotes the translocation of Drp1 from the cytosol to the mitochondrial outer membrane, whereas the phosphorylation of Serine 637 (Ser637) reverses this process 35, 36. We found that p-Drp1^S616^, but not p-Drp1^S637^, was highly expressed in the epithelial cells of mice with PVL and TNF-α-treated GES-1 cells. However, *shFADD* or Nec-1 reduced the Ser616 phosphorylation of Drp1, and *shFADD* decreased the TNF-α-induced mitochondrial translocation of Drp1 in GES-1 cells (**Figure 4A, 4B**). Mitochondrial division inhibitor 1 (Mdivi-1) attenuated mucosal injury and 4-HNE expression in mice with PVL (**Figure 4C**). Mdivi-1 alleviated mtROS levels, partly restored abnormal or damaged mitochondria and increased ATP concentrations and cell viability in epithelial cells from mice with PVL (**Figure 4D, 4E**). Furthermore, Mdivi-1 decreased epithelial 4-HNE levels, ROS and MDA levels, and downregulated cleaved caspase-1 and NLRP3 expression but did not affect the levels of FADD, RIPK1 or caspase-8 in epithelial cells from mice with PVL (**Figure 4F**). Cytosolic and mitochondrial Ca^2+^ fluorescence staining revealed that in TNF-α-treated GES-1 cells, a higher cytosolic Ca^2+^ concentration paralleled the increase in mitochondrial Ca^2+^ (**Figure 4G**). The cytosolic and mitochondrial Ca^2+^ concentrations were greater in the TNF-α-treated GES-1 cells than in the control cells (as vehicle), and Mdivi-1 reversed these increases (**Figure 4G**). Taken together, these findings indicate that the FADDosome enhances Drp1/Fis1-dependent mitochondrial fission to induce gastric epithelial mitochondrial dysfunction and oxidative stress in PHG (**Figure 4H**).

### NOX2 enhances FADDosome-induced gastric epithelial mitochondrial fission and dysfunction in PHG

**Figure 5:**
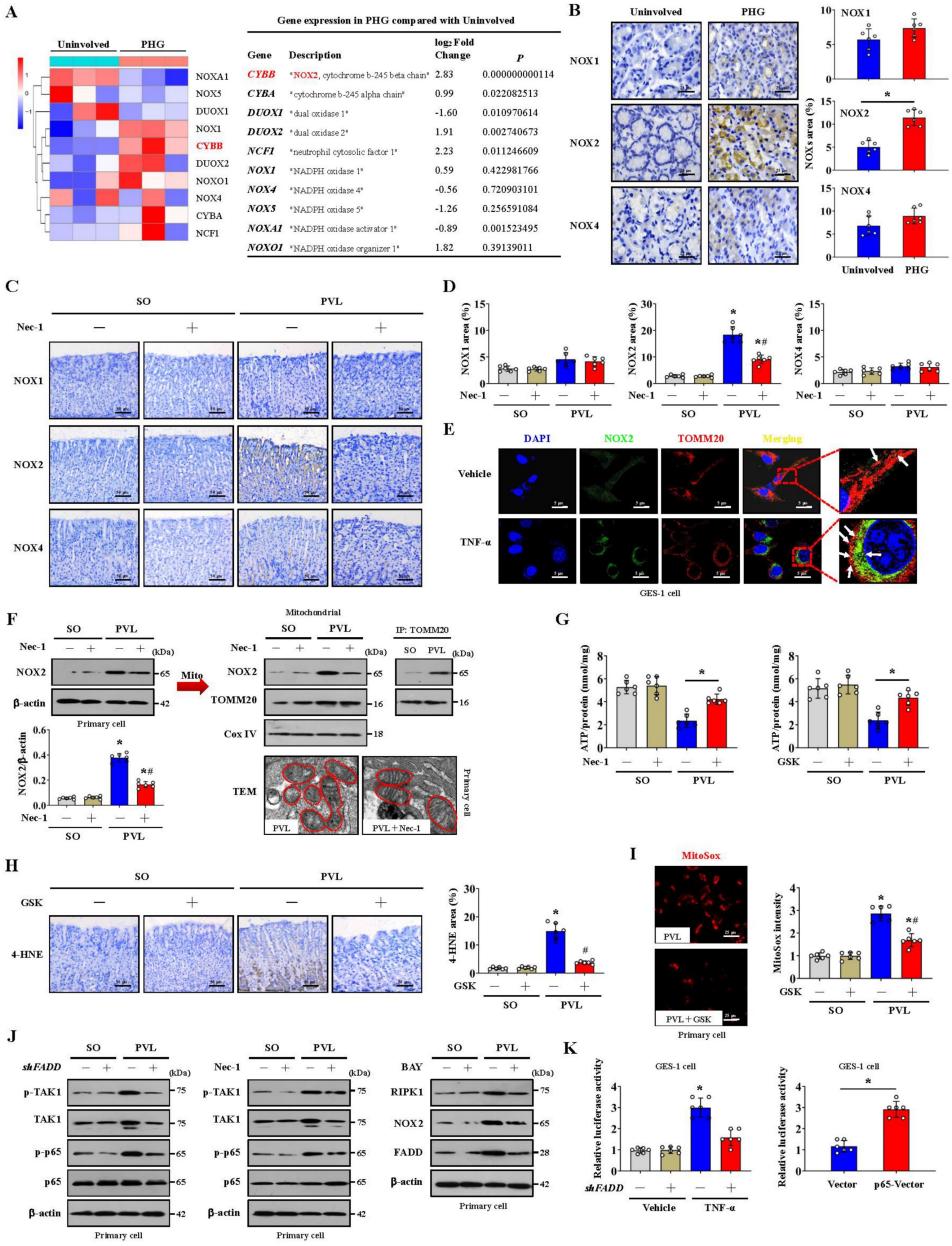
** NOX2 enhances FADDosome-induced gastric epithelial mitochondrial fission and dysfunction in PHG.** (A) Two-dimensional hierarchical clustering results for the enzyme NADPH oxidase (NOX) family were presented for the comparison between PHG patients and healthy volunteers (n = 3 per group). The fold changes and *P* values of the indicated mRNAs in PHG tissues relative to those in normal (Uninvolved) tissues from the microarray experiment were also presented. (B) NOX1, NOX2 and NOX4 IHC staining (brown) showed that NOX2, but not NOX1 or NOX4, was upregulated in the gastric mucosal samples of PHG patients, and the NOX1, NOX2 and NOX4 areas were also analyzed. n = 6 per group. *P < 0.05. (C) Representative images of IHC staining (brown) for NOX1, NOX2 and NOX4 in mouse models revealed that Nec-1 (an inhibitor of RIPK1) repressed NOX2 expression in mice with PVL. n = 6 per group. (D) The NOX1, NOX2 and NOX4 areas detected by IHC staining in (C) were presented. n = 6 per group. *P < 0.05 versus SO mice; #*P* < 0.05 versus mice with PVL without Nec-1 treatment. (E) The colocalization of TOMM20 (red) and NOX2 (green) determined by confocal imaging in GES-1 cells confirmed that TNF-α increased the mitochondrial localization of NOX2 (white arrows indicate colocalization). Nuclei (blue) were counterstained with DAPI. (F) The levels of and interaction between NOX2 and TOMM20 were determined by western blotting, and IP showed that Nec-1 decreased the epithelial expression of total and mitochondrial NOX2 in the primary epithelial cells of mice with PVL. TEM also revealed that Nec-1 partly normalized the abnormal or damaged mitochondria in the mice with PVL. n = 6 per group. *P < 0.05 versus SO mice; #*P* < 0.05 versus mice with PVL without Nec-1 treatment. (G) The ATP concentration in the mouse models verified that Nec-1 and GSK2795039 (GSK, a NOX2 inhibitor) improved the production of ATP in mice with PVL. n = 6 per group. *P < 0.05. (H) Representative 4-HNE IHC staining (brown) and quantification analysis in SO mice and mice with PVL suggested that GSK2795039 (GSK) reduced the level of 4-HNE in mice with PVL. n = 6 per group. *P < 0.05 versus SO mice; #*P* < 0.05 versus mice with PVL without GSK2795039 treatment. (I) Representative MitoSox staining (red) and the MitoSox intensity in mouse model revealed that GSK2795039 alleviated mitochondrial ROS (mtROS) accumulation in the primary epithelial cells of mice with PVL. n = 6 in each group. *P < 0.05 versus SO mice; #*P* < 0.05 versus mice with PVL without GSK2795039 treatment. (J) Related protein levels in primary epithelial cells from mouse models treated with the indicated agents (*shFADD*, Nec-1 or BAY) showed interactions among TAK1, NF-κBp65, FADD and NOX2 signaling. (K) GES-1 cells were transfected with either the control vector or *shFADD* and then treated with TNF-α or vehicle, and the *NOX2* luciferase reporter activities were presented (left panel). *P < 0.05 versus the vehicle group and TNF-α-treated cells transfected with *shFADD*. The *NOX2* luciferase reporter activities in GES-1 cells transfected with *pcDNA3.1-p65*-vector or *pcDNA3.1*-vector were also shown (right panel), *P < 0.05.

Multiple NOXs were modulated in PHG patients compared to healthy people; in particular, upregulated *NOX2* (*CYBB*) was observed in the gastric mucosa of PHG patients (**Figure 5A**). IHC staining confirmed that NOX2, rather than NOX1 or NOX4, was upregulated in the gastric mucosa of mice with PVL and PHG patients (**Figure 5B-5D**), and Nec-1 repressed NOX2 expression in the mice with PVL (**Figure 5C, 5D**). The mitochondrial localization of NOX2 was increased in GES-1 cells by TNF-α (**Figure 5E**). Moreover, TEM analysis and proteins detection revealed that Nec-1 partly relieved the abnormal or damaged mitochondria and decreased the epithelial expression of total NOX2, particularly the expression of mitochondrial NOX2, in the mice with PVL (**Figure 5F**). The production of ATP in the gastric mucosa of the mice with PVL was improved by Nec-1 and the NOX2 inhibitor GSK2795039 (**Figure 5G**). Moreover, GSK2795039 reduced the levels of 4-HNE and mtROS (as detected by MitoSox) in the mucosa and epithelial cells of the mice with PVL (**Figure 5H, 5I**). NOX2 can increase ROS levels to cause mitochondrial fission and reduce mitochondrial respiration and therefore promote glycolysis in some pathological processes 37. We also investigated whether NOX2 affects mitochondrial fission in PHG. Western blotting demonstrated that GSK2795039 decreased the levels of Drp1 and Fis1 in epithelial cells from the mice with PVL without affecting the status of FADD or caspase-8 (**Figure S3A**). Mdivi-1 did not affect NOX2 expression in epithelial cells from the mice with PVL (**Figure S3B**), suggesting that Drp1-dependent mitochondrial fission did not regulate NOX2 signaling. Furthermore, Mito-Tracker staining revealed an increase in mitochondrial fragmentation in the isolated epithelial cells of the mice with PVL, which was obviously alleviated by GSK2795039 (**Figure S3C**). These data suggested that NOX2 modulates Drp1-dependent mitochondrial fission in PHG.

Our previous studies have identified NF-κBp65 phosphorylation/activation in gastric mucosal samples of PHG 4, 5. TAK1 is a quintessential kinase that positively regulates inflammation through NF-κB signaling cascades 38. Increased levels of both phosphorylated TAK1 (p-TAK1) and phosphorylated NF-κBp65 (p-NF-κBp65) were found in the mice with PVL, while *shFADD* repressed these changes (**Figure 5J**). TAK1 mediates the phosphorylation of RIPK1 to stabilize RIPK1 in an NF-κB signaling state 13, 38, and western blotting revealed that Nec-1 decreases NF-κBp65 phosphorylation/activation without affecting p-TAK1 status (**Figure 5J**). Furthermore, NF-κBp65 activation in the mice with PVL not only increased NOX2 levels but also promoted FADD and RIPK1 expression, and these effects were reversed by BAY (NF-κB inhibitor) (**Figure 5J**). By utilizing a luciferase assay in GES-1 cells, we found that both TNF-α treatment and *p65*-vector transfection activated the *NOX2* reporter, whereas *shFADD* transfection blunted the activation of the *NOX2* reporter by TNF-α (**Figure 5K**). These data elucidated the molecular mechanism of the interaction between p-TAK1 and RIPK1 in the activation of the NF-κBp65 pathway, which could promote the transcription of *NOX2* (*CYBB*). In summary, NOX2 enhances FADDosome-induced epithelial mitochondrial fission and dysfunction in PHG.

### FADDosome-induced mitochondrial dysfunction influences the metabolic profile by altering glycolysis in PHG

**Figure 6:**
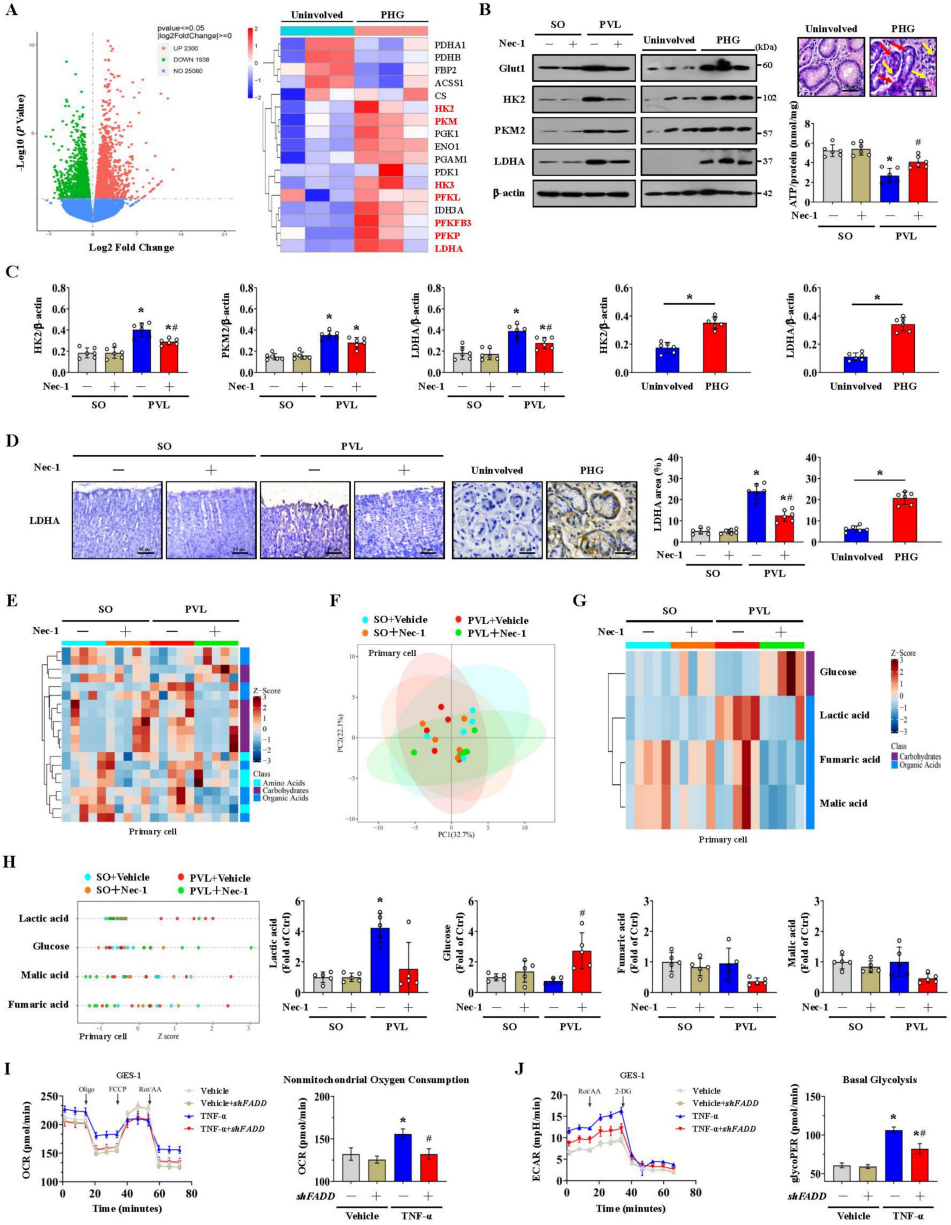
** FADDosome-induced mitochondrial dysfunction influences the metabolic profile by altering glycolysis in PHG.** (A) Volcano and heatmap plots showing the results from the analysis of metabolism-related gene expression suggested that glycolysis-encoding genes were upregulated in the gastric mucosal samples of PHG patients, n = 3 in each group. (B) Western blotting showed that the protein levels of Glut1, HK2, PKM2 and LDHA were increased in both PHG patients and mice with PVL but decreased by Nec-1 in mice with PVL. H&E staining revealed mucosal congestion (yellow arrow) and epithelial injury (red arrow) in the PHG sections. ATP concentrations in mouse models suggested that Nec-1 treatment enhanced ATP production in mice with PVL. n = 6 per group. *P < 0.05 versus SO mice; #*P* < 0.05 versus mice with PVL without Nec-1 treatment. (C) Ratios of densitometric units of normalized HK2/β-actin, PKM2/β-actin and LDHA/β-actin were determined from western blotting (n = 6 per group). *P < 0.05 versus SO mice or the uninvolved group; #*P* < 0.05 versus mice with PVL without Nec-1 treatment. (D) Representative IHC staining (brown) and quantitative analysis of LDHA revealed that LDHA levels were increased in both PHG patients and mice with PVL and that Nec-1 repressed LDHA levels in mice with PVL. n = 6 in each group. *P < 0.05 versus SO mice or the uninvolved group; #*P* < 0.05 versus mice with PVL without Nec-1 treatment. (E) Heatmap of the results of an untargeted metabolomics strategy (for amino acids, carbohydrates and organic acids) applied to primary epithelial cells from SO and PVL model mice treated with or without Nec-1. n = 5 per group. The colors represent the levels of the metabolites from low to high. (F) A PLS-DA score plot of five independent preparations of primary epithelial cells suggested that there was a significant alteration in the metabolites among the indicated groups, n = 5 per group. (G) Heatmap of metabolic alterations in glucose, lactic acid, fumaric acid and malic acid suggested that they were potential metabolic biomarkers among the indicated four groups. n = 5 per group. (H) The relative levels of glucose, lactic acid, fumaric acid and malic acid were presented via a Z score plot (left panel) and boxplot (right panel). n = 5 per group. *P < 0.05 versus SO mice; #*P* < 0.05 versus mice with PVL without Nec-1 treatment. (I) Mitochondrial respiration (for oxygen consumption rate [OCR]) analysis revealed that TNF-α treatment of GES-1 cells influenced mitochondrial respiration and increased nonmitochondrial oxygen consumption, which could be reversed by *FADD* knockdown (*shFADD* transfection). n = 6 in each group. *P < 0.05 versus vehicle group; #*P* < 0.05 versus TNF-α-treated cells without *shFADD* transfection. (J) Glycolytic rate (for extracellular acidification rate [ECAR]) detection showed that glycolysis and basal glycolysis were increased in response to TNF-α treatment but were rescued by *shFADD*. n = 6 in each group. *P < 0.05 versus vehicle group; #*P* < 0.05 versus TNF-α-treated cells without *shFADD* transfection.

Glycolysis is the other core energy-producing pathway in cells. We examined the transcriptomes of gastric mucosal tissues for the assessment of metabolic profiles. The glycolysis-encoding genes *hexokinase-2* (*HK2*), *hexokinase-3* (*HK3*), *lactate dehydrogenase A* (*LDHA*), *pyruvate kinase M* (*PKM*), *phosphofructokinase L* (*PFKL*), *6-phosphofructo-2-kinase fructose-2,6-biphosphatase 3* (*PFKFB3*) and *phosphofructokinase P* (*PFKP*) were upregulated in the gastric mucosa of PHG patients (**Figure 6A**). The protein levels of glucose transporter 1 (Glut1), HK2, PKM2 and LDHA were increased in both PHG patients and mice with PVL, and the upregulation of HK2 and LDHA rather than PKM2 was decreased by Nec-1 in mice with PVL (**Figure 6B, 6C**). Moreover, the gastric mucosa of PHG patients exhibited apparent mucosal congestion and epithelial injury, and mice with PVL exhibited decreased ATP production, whereas Nec-1 rescued these changes (**Figure 6B**). PKM2 combines with other key enzymes involved in glycolysis, such as pyruvate dehydrogenase kinase 1 (PDK1), LDHA, and Glut1. PKM2 can also function as a coactivator that stimulates hypoxia-inducible factor 1 (HIF-1) transactivation of target genes encoding PDK1, LDHA, and Glut1 in cancer cells. PKM2 exists as a monomer, dimer and tetramer, and its enzymatic activity occurs in a monomer-dimer-tetramer equilibrium that is complexly regulated to allow cells to adapt to different physiological states 39-41. To test whether PKM2 is involved in FADDosome-regulated glycolysis in PHG, we detected its downstream proteins following *shFADD* transfection and found that *FADD* knockdown by *shFADD* repressed LHDA upregulation in mice with PVL without affecting the levels of PKM2 isomeric forms (monomer, dimer or tetramer), Glut1 or PDK1 (**Figure S4A**). Moreover, *in vitro* experiments revealed that *shFADD* decreased the upregulation of LHDA and Glut1 but did not alter the expression of the PKM2 isomeric form or PDK1 in TNF-α-treated GES-1 cells (**Figure S4B**). These data indicate that *FADD* knockdown does not contribute to PKM2-related signaling in PHG.

The mucosal LDHA levels were increased in both PHG patients and mice with PVL, and these increases were inhibited by Nec-1 (**Figure 6D**). We next investigated gastric epithelial metabolism using an untargeted metabolomics strategy (**Figure 6E**). A partial least squares discriminant analysis (PLS-DA) model suggested that the metabolites significantly differed between the SO and PVL groups treated with or without Nec-1 (**Figure 6F**), and four metabolites related to energy metabolism, glucose, lactic acid, fumaric acid and malic acid, were suggested to be potential metabolic alteration biomarkers among the indicated four groups (**Figure 6G**). Nec-1 caused apparent alterations in the levels of energy-related metabolites in these cell extracts, and the changes included an increase in glucose and a decrease in lactic acid (**Figure 6G, 6H**). By detecting the oxygen consumption rate (OCR, an indicator of mitochondrial respiration), we found that increased nonmitochondrial oxygen consumption and proton leakage, as well as reduced maximum respiration, were induced by TNF-α in GES-1 cells and were reversed by *FADD* knockdown (*shFADD* transfection) (**Figure 6I, Figure S5A**). The glycolytic rate determined by the extracellular acidification rate (ECAR) was also analyzed, and basal glycolysis and compensatory glycolysis were found to increase in response to TNF-α treatment, which were decreased by *shFADD* (**Figure 6J, Figure S5B**). These data indicate that FADD is involved in the alteration of mitochondrial respiration and the glycolytic rate and that* FADD* knockdown could enhance mitochondrial respiration and decrease glycolysis.

### The alteration of glycolysis associated with dysfunction of the mitochondrial electron transport chain contributes to oxidative stress in the PHG

**Figure 7:**
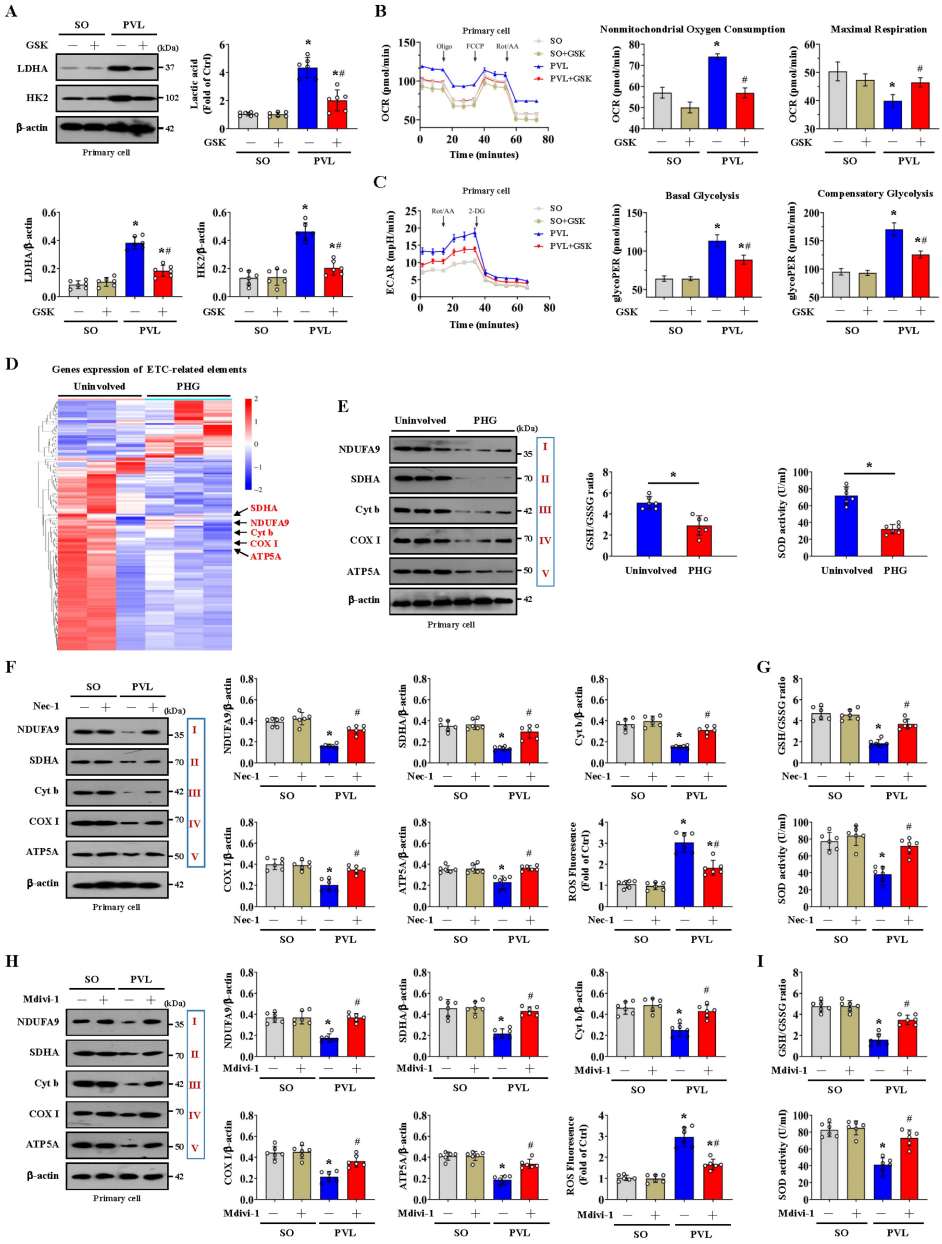
** The alteration of glycolysis associated with dysfunction of the mitochondrial electron transport chain contributes to oxidative stress in the PHG.** (A) Western blotting showed that the protein levels of LDHA and HK2 were increased in mice with PVL but were decreased by GSK2795039 (GSK, a NOX2 inhibitor) in mice with PVL. The ratios of the normalized LDHA/β-actin and HK2/β-actin densitometric units and the relative level of lactic acid were also analyzed. n = 6 per group. *P < 0.05 versus SO mice; *#P* < 0.05 versus mice with PVL not treated with GSK2795039. (B) Oxygen consumption rate (OCR) analysis of primary epithelial cells isolated from mouse models suggested that increased nonmitochondrial oxygen consumption and reduced maximum respiration were present in mice with PVL but were reversed by GSK2795039. n = 6 in each group. *P < 0.05 versus SO mice; #*P* < 0.05 versus mice with PVL without GSK2795039 treatment. (C) Examination of the glycolytic rate (for the extracellular acidification rate [ECAR]) showed that GSK2795039 treatment reduced basal and compensatory glycolysis in mice with PVL. n = 6 in each group. *P < 0.05 versus SO mice; #*P* < 0.05 versus mice with PVL without GSK2795039 treatment. (D) Two-dimensional hierarchical clustering results of the genes of electron transport chain (ETC)-related elements between PHG patients (as PHG) and healthy volunteers (as Uninvolved) (n = 3 per group). (E) Western blotting analysis (left panel) of protein levels of representative ETC complex subunits in the primary epithelial cells of PHG patients and healthy volunteers (as Uninvolved) revealed that the levels of the mitochondrial complex I, II, III, IV and V subunits were decreased in PHG patients. The activity of the antioxidant enzyme SOD and the ratio of reduced (GSH) to oxidized (GSSG) states (GSH/GSSG) detected by assay kits (right panel) were found to be decreased in PHG. n = 6 per group. *P < 0.05. (F) Western blotting analysis of representative ETC complex subunits from primary epithelial cells isolated from mouse models suggested that Nec-1 (an inhibitor of RIPK1) mitigated the defects in ETC subunit expression and decreased ROS levels in mice with PVL. The ratios of the normalized NDUFA9/β-actin, SDHA/β-actin, Cyt b/β-actin, COX I/β-actin and ATP5A/β-actin densitometric units and the levels of ROS were also analyzed. n = 6 per group. *P < 0.05 versus SO mice; *#P* < 0.05 versus mice with PVL without Nec-1 treatment. (G) SOD activity and the GSH/GSSG ratio, as detected by assay kits, were inhibited in mice with PVL but were restored by Nec-1 treatment. n = 6 per group. *P < 0.05 versus SO mice; *#P* < 0.05 versus mice with PVL without Nec-1 treatment. (H) Western blotting analysis and quantification of protein levels for representative ETC complex subunits from primary epithelial cells isolated from mouse models showed that Mdivi-1 reversed the decrease in mitochondrial complex I, II, III, IV and V subunit levels and reduced ROS levels in mice with PVL. The ratios of the normalized NDUFA9/β-actin, SDHA/β-actin, Cyt b/β-actin, COX I/β-actin and ATP5A/β-actin densitometric units and the levels of ROS were also presented. n = 6 per group. *P < 0.05 versus SO mice; *#P* < 0.05 versus mice with PVL without Mdivi-1 treatment. (I) Decreases in SOD activity and the GSH/GSSG ratio detected by assay kits were found in mice with PVL but were reversed by Mdivi-1 treatment. n = 6 per group. *P < 0.05 versus SO mice; *#P* < 0.05 versus mice with PVL without Mdivi-1 treatment.

NOX2 is intimately related to mitochondrial efficiency and glycolysis. NOX2 can increase ROS levels to cause mitochondrial fission and reduce mitochondrial respiration and therefore promote glycolysis in some pathological processes 37. We also investigated whether NOX2 affects glycolytic activity in PHG. The expression of the glycolytic proteins LDHA and HK2 and the lactic acid concentration were obviously increased in the epithelial cells of the mice with PVL, while the expression of LDHA and HK2 and the lactic acid concentration were decreased by the NOX2 inhibitor GSK2795039 (**Figure 7A**). By detecting the OCR, we found increased nonmitochondrial oxygen consumption and reduced maximum respiration were existed in the primary epithelial cells of mice with PVL, and this change was reversed by GSK2795039 (**Figure 7B**). The rate of glycolysis, determined by the ECAR, was also measured, and basal and compensatory glycolysis were found to be increased in mice with PVL but were repressed by GSK2795039 (**Figure 7C**). These data indicated that NOX2 participated in the alteration of mitochondrial respiration and the glycolytic rate in PHG, while GSK2795039 enhanced mitochondrial respiration and decreased glycolysis to provide protection against NOX2-mediated mitochondrial dysfunction in PHG.

Mitochondrial ATP generation and ROS production are intimately related to the function of the mitochondrial electron transport chain (ETC). Nicotinamide adenine dinucleotide (NADH) and flavin adenine dinucleotide (FADH_2_), which are supplied by glycolysis, translocate to the ETC, each of which can donate a pair of electrons to the ETC 42. The physiological function of ETC-generated mtROS is required for homeostatic signaling in the organelle, and antioxidant systems contribute to the regulation of mtROS concentration and redox species. ETC dysfunction leads to the premature leakage of electrons from complexes I, II, and III to enhance mtROS generation to aggravate various pathophysiological processes in the organelle 42. To explore whether a defect in the ETC contributes to mtROS production under altered glycolysis status, we examined the transcriptomes of gastric mucosal tissues to assess the ETC profiles and found that multiple ETC-related elements were modulated between PHG patients and uninvolved volunteers (**Figure 7D**). Western blotting analysis of representative ETC complex subunits revealed that the expression levels of NADH ubiquinone oxidoreductase subunit A9 (NDUFA9), succinate dehydrogenase complex flavoprotein subunit A (SDHA), cytochrome b (Cyt b), cyclooxygenase I (COX I) and alpha subunit of ATP synthase (ATP5A) were decreased in both PHG patients and mice with PVL (**Figure 7E, 7F, 7H**). The activity of the antioxidant enzyme superoxide dismutase (SOD) and the ratio of reduced (GSH, glutathione) to oxidized (GSSG, glutathione disulfide) states (GSH/GSSG) were also decreased in both PHG patients and mice with PVL (**Figure 7E, 7G, 7I**). Moreover, inhibition of RIPK1 by Nec-1 (**Figure 7F, 7G**) or blockade of mitochondrial fission by Mdivi-1 (**Figure 7H, 7I**) reversed the defects in the expression of the abovementioned ETC complex subunits, decreased ROS levels, and enhanced SOD activity and GSH/GSSG ratio in mice with PVL. These results demonstrate that the alterations in glycolysis and antioxidant enzyme activity associated with ETC dysfunction contribute to oxidative stress and mtROS production in PHG.

### Mitochondrial dysfunction-induced oxidative stress activates the epithelial NLRP3 inflammasome to promote epithelial pyroptosis and mucosal injury

NLRP3 senses mitochondrial dysfunction and may explain the frequent association of mitochondrial damage with diseases 25, 43. NLRP3 activation is closely associated with the initiation of pyroptosis, in which NLRP3 recruits and binds to ASC complexes to lead to autocleavage and activation of procaspase-1, after which cleaved caspase-1 results in the cleavage of GSDMD and activation of inactive cytokines such as IL-18 and IL-1β to induce pyroptosis 26.

**Figure 8:**
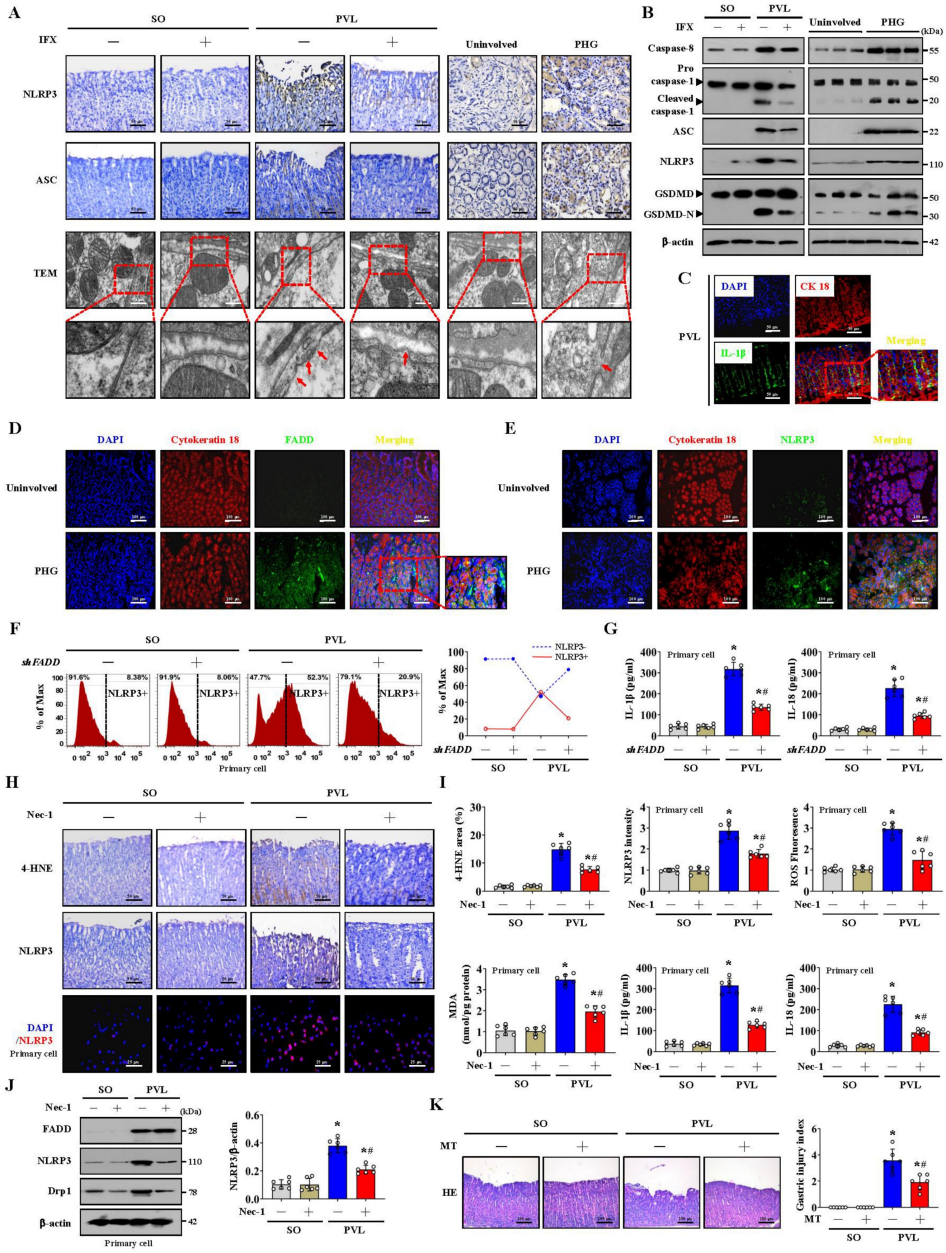
** Mitochondrial dysfunction-induced oxidative stress activates the epithelial NLRP3 inflammasome to promote epithelial pyroptosis and mucosal injury.** (A) Representative IHC staining for NLRP3 and ASC in the indicated groups revealed that the levels of these proteins were increased in both PHG patients and mice with PVL and that IFX repressed the expression in mice with PVL. Membrane pore formation features (red arrows) detected by TEM also showed increased membrane pore formation in the gastric epithelial cells of PHG patients and mice, and IFX decreased this formation in mice with PVL. n = 6 per group. (B) Western blotting for caspase-8, procaspase-1, cleaved caspase-1, ASC, NLRP3, GSDMD and GSDMD-N in the indicated groups demonstrated that these proteins were upregulated in PHG patients and mice and that was downregulated by IFX in mice with PVL. n = 6 per group. (C) Double IF staining showed enhanced IL-1β (green) production around epithelial cells (CK18, cytokeratin 18, red) in mice with PVL. Nuclei (blue) were counterstained with DAPI. (D and E) Double IF staining of CK18 (red) and FADD (green), CK18 (red) and NLRP3 (green) in the uninvolved and PHG groups suggested that both FADD and NLRP3 were located in the mucosal epithelium of the PHG. Nuclei (blue) were counterstained with DAPI. (F) The percentage of NLRP3+ cells was measured by flow cytometry analysis in primary cells isolated from mouse models treated with or without *shFADD*, and *shFADD* blunted the increase in NLRP3+ epithelial cells in mice with PVL. n = 6 per group. (G) The concentrations of IL-1β and IL-18 in the medium of primary epithelial cells in the indicated groups determined by ELISAs revealed that *shFADD* decreased the production of IL-1β and IL-18 in mice with PVL. n = 6 per group. *P < 0.05 versus SO mice; *#P* < 0.05 versus mice with PVL not treated with* shFADD*. (H) Representative images of 4-HNE and NLRP3 IHC staining (brown) of gastric sections and NLRP3 IF staining (red) of primary cells from mouse models showed that Nec-1 reduced the levels of 4-HNE and NLRP3 in mice with PVL. Nuclei (blue) were counterstained with DAPI. (I) Quantitative analysis of 4-HNE area, NLRP3 intensity, and primary epithelial ROS, MDA, IL-1β and IL-18 levels suggested that Nec-1 reduced their levels in mice with PVL. n = 6 per group. *P < 0.05 versus SO mice; *#P* < 0.05 versus mice with PVL without Nec-1 treatment. (J) Western blotting for FADD, NLRP3 and Drp1 in the indicated groups suggested that Nec-1 repressed NLRP3 and Drp1 upregulation without affecting FADD levels. The ratio of densitometric units of the normalized NLRP3/β-actin ratio was also calculated. *P < 0.05 versus SO mice; *#P* < 0.05 versus mice with PVL without Nec-1 treatment. (K) Representative photographs of H&E staining and quantitative analysis of gastric injury index in the indicated sections verified that the ROS scavenger Mito-TEMPO (MT) relieved gastric mucosal injury in mice with PVL. n = 6 in each group, *P < 0.05 versus SO mice; *#P* < 0.05 versus mice with PVL without MT treatment.

Histological staining revealed that NLRP3 and ASC levels were increased in both PHG patients and mice with PVL, while IFX repressed NLRP3 and ASC levels in mice with PVL (**Figure 8A, Figure S6A**). Increased membrane pore formation was found in the gastric epithelial cells of PHG patients and mice with PVL, and this increase was reduced by IFX (**Figure 8A, Figure S6A**). Western blotting analysis revealed that the levels of caspase-8, cleaved caspase-1, ASC, NLRP3 and GSDMD-N were obviously greater in the PHG patients and mice with PVL than in the controls, while IFX decreased the levels of these proteins in the mice with PVL (**Figure 8B, Figure S6B, S6C**). Costaining revealed that IL-1β production around the epithelia was apparently enhanced in the mice with PVL (**Figure 8C**). FADD, NLRP3 and CK18 were also colocalized in the epithelial cells of the PHG group, suggesting that both FADD and NLRP3 are involved in gastric mucosal epithelial injury in PHG (**Figure 8D, 8E**). Consistently, 52.3% NLRP3+ epithelial cells were observed in mice with PVL compared to 8.38% NLRP3+ epithelial cells in SO mice without *FADD* knockdown according to flow cytometric analysis, and this percentage was reduced to 20.9% after *FADD* knockdown in mice with PVL (**Figure 8F**). Moreover, knocking down* FADD* decreased the production of IL-1β and IL-18 in mice with PVL (**Figure 8G**). Nec-1 attenuated gastric mucosal damage and reduced the levels of 4-HNE, MDA, ROS, and NLRP3 and the production of IL-1β and IL-18 in mice with PVL (**Figure 8H, 8I**). Moreover, Nec-1 suppressed NLRP3 and Drp1 upregulation without affecting FADD levels (**Figure 8J**). As a mitochondrion-targeted antioxidant, the ROS scavenger Mito-TEMPO (MT) was demonstrated to relieve gastric mucosal injury and reduce ASC and NLRP3 levels in mice with PVL, and MT could also restore epithelial cell viability in mice with PVL (**Figure 8K, Figure S7A, S7B**). Moreover, MT suppressed NLRP3, cleaved caspase-1 and GSDMD-N levels and decreased IL-1β and IL-18 production in the epithelial cell of mice with PVL without affecting FADD/RIPK1/caspase-8 elements (**Figure S7C, S7D**), which highlights the protective role of MT in NLRP3 inflammasome activation-mediated pyroptosis and mucosal injury in PHG. Furthermore, histological staining and proteins analysis revealed that GSK2795039 alleviated NLRP3 inflammasome activation in mice with PVL (**Figure S7E, S7F**). In summary, the data indicate that mitochondrial dysfunction-induced oxidative stress activates the epithelial NLRP3 inflammasome to promote epithelial pyroptosis and mucosal injury in PHG.

### The scaffolding function of caspase-8 in the FADDosome regulates mitochondrial dysfunction-induced NLRP3 inflammasome activation to exacerbate epithelial pyroptosis

**Figure 9:**
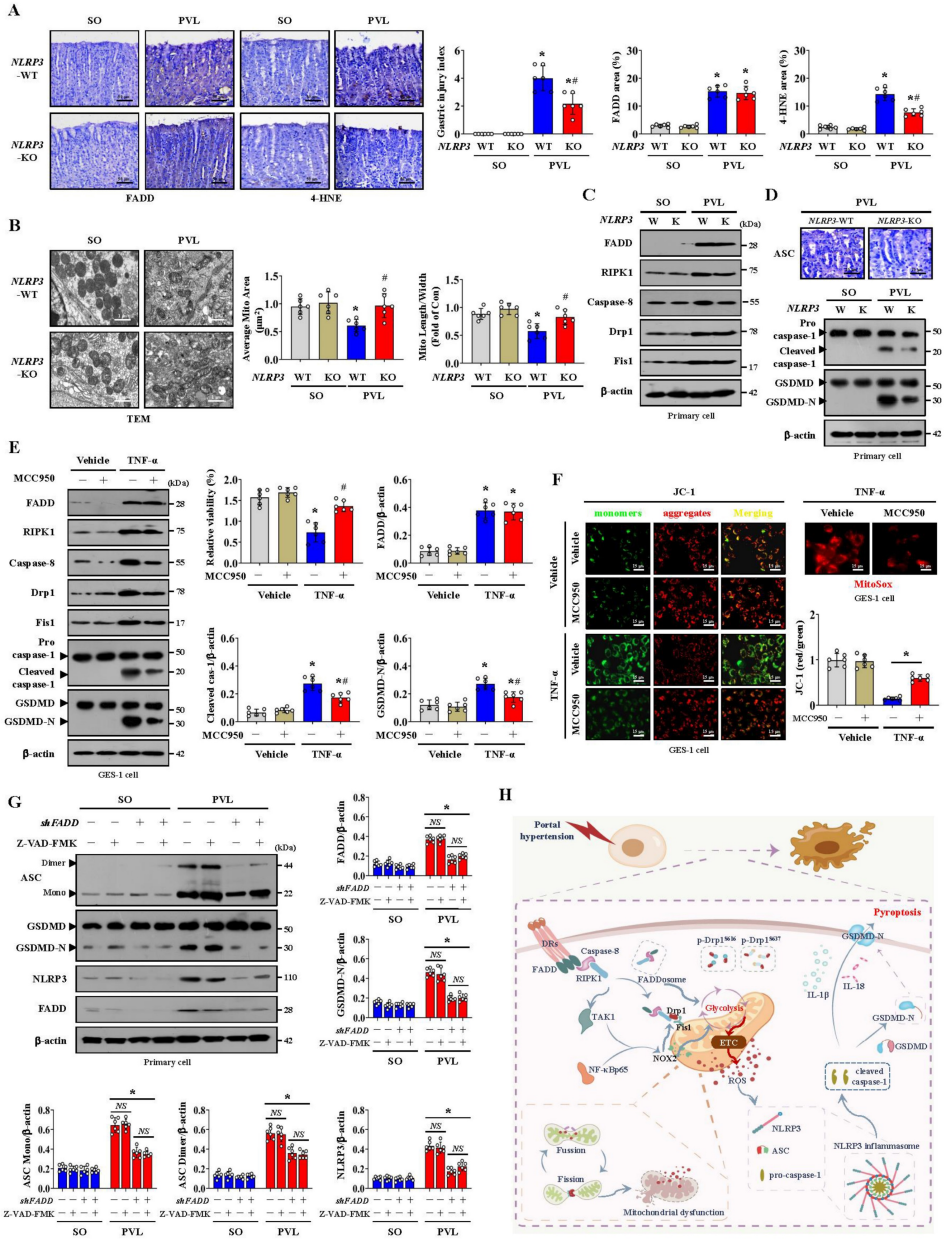
** The scaffolding function of caspase-8 in the FADDosome regulates mitochondrial dysfunction-induced NLRP3 inflammasome activation to exacerbate epithelial pyroptosis.** (A) IHC staining showing the expression of FADD (brown) and 4-HNE (brown) in* NLRP3* wild-type (*NLRP3*-WT) and *NLRP3* knockout (*NLRP3*-KO) mouse models, and revealed that enhanced 4-HNE levels were observed in *NLRP3*-WT mice with PVL compared with those in *NLRP3*-KO mice, and no significant difference in FADD expression was observed between them. The gastric injury index, the FADD and 4-HNE areas were also analyzed. n = 6 per group. *P < 0.05 versus SO mice; #*P* < 0.05 versus PVL *NLRP3*-WT mice. (B) Representative TEM images and quantification of the average mitochondrial area and mitochondrial length/width index demonstrated that *NLRP3* deficiency could partly reverse this mitochondrial damage. n = 6 in each group. *P < 0.05 versus SO mice;* #P* < 0.05 versus PVL-treated *NLRP3*-WT mice. (C) Western blotting for FADD, RIPK1, caspase-8, Drp1 and Fis1 in primary isolated epithelial cells from the indicated groups showed that *NLRP3* deficiency did not affect the expression of these proteins in mice with PVL. n = 6 in each group. (D) ASC IHC staining (brown) of mouse models revealed that ASC was inhibited in PVL-treated *NLRP3*-KO mice. The cleaved caspase-1 and GSDMD-N levels assessed by western blotting demonstrated that their levels were decreased in PVL-treated *NLRP3*-KO mice. n = 6 in each group. (E) The levels of FADD, RIPK1, caspase-8, Drp1, Fis1, procaspase-1, cleaved caspase-1, GSDMD and GSDMD-N in vehicle- or TNF-α-treated GES-1 cells by western blotting showed that FADD/caspase-8/RIPK1 signaling and Drp1/Fis1 expression were not influenced by MCC950 (a selected NLRP3 inhibitor) in TNF-α-treated cells. The cell viability and the ratios of densitometric units of normalized FADD/β-actin, cleaved caspase-1/β-actin and GSDMD-N/β-actin were further determined. n = 6 per group. *P < 0.05 versus the vehicle group; #*P* < 0.05 versus TNF-α-treated GES-1 cells without MCC950 treatment. (F) Mitochondrial membrane potential was detected by JC-1 staining, and mitochondrial ROS levels were examined by MitoSox (red), which showed that MCC950 restored the mitochondrial membrane potential and decreased mtROS production in TNF-α-treated GES-1 cells. The histogram shows the quantification of JC-1 staining. n = 6 in each group. *P < 0.05. (G) The levels of ASC (dimer and monomer), GSDMD, GSDMD-N, NLRP3 and FADD in primary epithelial cells from the indicated groups determined by western blotting showed that the pan-caspase inhibitor Z-VAD-FMK did not affect the levels of these proteins in the primary epithelial fractions of mice with PVL transfected with *shFADD*. The ratios of densitometric units of normalized FADD/β-actin, GSDMD-N/β-actin, ASC monomer/β-actin, ASC dimer/β-actin and NLRP3/β-actin were determined. n = 6 per group. *P < 0.05. *NS*, not significant. (H) Schematic diagram of FADDosome-regulated mitochondrial fission and dysfunction in NLRP3 inflammasome-mediated epithelial pyroptosis and mucosal injury in PHG.

The PVL-induced PHG models established by generating* NLRP3*-WT and *NLRP3*-KO mice were used. Marked gastric injury with mucosal erosion and enhanced 4-HNE levels were observed in *NLRP3*-WT mice with PVL compared with those in *NLRP3*-KO mice, and no significant difference in FADD expression was observed between the PVL-treated *NLRP3*-WT and PVL-treated *NLRP3*-KO mice, although the FADD expression was enhanced in both of them (**Figure 9A**). A fragmented mitochondrial morphology and disruption of membrane integrity with broken or absent cristae were aggravated in the *NLRP3*-WT mice with PVL, and *NLRP3* deficiency (*NLRP3*-KO) could partly reverse this mitochondrial damage (**Figure 9B**). No significant differences in FADD/caspase-8/RIPK1 signaling, Drp1 or Fis1 expression were observed between the PVL-treated *NLRP3*-WT and PVL-treated *NLRP3*-KO mice (**Figure 9C, Figure S8A**), while ASC, cleaved caspase-1 and GSDMD-N were obviously inhibited in the PVL-treated *NLRP3*-KO mice compared with those in PVL-treated *NLRP3*-WT mice (**Figure 9D, Figure S8B**). The primary cell viability of the PVL-treated *NLRP3*-WT mice was markedly decreased, while that of the PVL-treated *NLRP3*-KO mice was increased (**Figure S8C**).

Consistently, no significant differences in FADD/caspase-8/RIPK1 signaling or Drp1 or Fis1 expression were observed between the TNF-α-treated GES-1 cells treated with MCC950 (a selected NLRP3 inhibitor) and those not treated with MCC950 (**Figure 9E**). Cleaved caspase-1 and GSDMD-N levels were lower in the TNF-α-treated cells following with MCC950 than in those not following with MCC950 (**Figure 9E**). Moreover, the cell viability of the TNF-α-treated cells was markedly decreased, while MCC950 treatment restored cell viability (**Figure 9E**). MCC950 prevented mitochondrial depolarization (decreased mitochondrial membrane potential) and reduced mtROS production in TNF-α-treated GES-1 cells (**Figure 9F**). These data indicate that NLRP3 exacerbates the epithelial pyroptosis and mucosal injury induced by mitochondrial dysfunction.

Several studies have revealed the contribution of caspase-8 catalytic activity to NLRP3 inflammasome activation and GSDMD cleavage 44. The pan-caspase inhibitor Z-VAD-FMK (which inhibits caspase-1 and caspase-8 activation) was utilized, and we found that the presence or absence of Z-VAD-FMK induced high amounts of polymerized ASC and NLRP3 associated with FADD and GSDMD-N in the epithelial fractions of mice with PVL not treated with *shFADD* (**Figure 9G**). Additionally, the abovementioned proteins in the epithelial fractions of the mice with PVL transfected with *shFADD* were not altered, regardless of whether Z-VAD-FMK was added (**Figure 9G**). The data indicate that the scaffolding function of caspase-8 but not its enzymatic activity is required in the FADDosome-regulated NLRP3 activation and GSDMD cleavage.

In summary, the FADDosome enhances mitochondrial dysfunction and influences the metabolic profile by altering the glycolytic profile to increase mtROS production to drive NLRP3 inflammasome activation, and thus the activation of NLRP3 inflammasome mediates gastric epithelial pyroptosis and mucosal injury in the PHG (**Figure 9H**).

## Discussion

Portal hypertensive gastropathy (PHG) occurs as the most common gastric mucosal injury among patients afflicted with cirrhotic or non-cirrhotic portal hypertension. Splanchnic blood flow, the distribution of mucosal blood, local disturbances and portal pressure have been examined to elucidate the underlying mechanisms in PHG 1-4. Connections of the perivascular lamina propria frequently present inflammatory cell infiltration and increased cytokine production 45. We found that, accompanied by gastric injury, TNF-α-related elements and their signal transducers were enhanced in the gastric mucosa of PHG. FADD is recruited by DRs to bind caspase-8 and dissociates from DRs to recruit RIPK1 to form the FADDosome 6, 8, 9. In the present study, elevated FADD in PHG patients and mice was positively correlated with the severity of gastric mucosal injury, and FADD recruited caspase-8 to form the FADD/caspase-8 complex in the gastric epithelial cells of PHG. The selective TNF-α inhibitor IFX attenuated mucosal injury, epithelial FADD expression and the interaction between FADD and caspase-8 in mouse PHG. Understanding the essential role of FADD in major biological processes, such as intracellular localization and the resulting associated activities, is critical. SUMOylated FADD was found to translocate to mitochondria, leading to Drp1-dependent mitochondrial fragmentation and caspase-independent necrosis during hypoxic and ischemic injury 46. The epithelial cells in PHG exhibited obvious mitochondrial swelling, fragmented morphology, and disruption of membrane integrity with broken or absent cristae associated with enhanced oxidative stress and mtROS production, while IFX or *FADD* knockdown attenuated the extent of mitochondrial fragmentation and oxidative stress, suggesting that the translocation of the FADDosome to the epithelial mitochondrial membrane contributes to mitochondrial dysfunction and oxidative stress in PHG.

TNF-α triggers the recruitment of RIPK1 to the FADD/caspase-8 complex, resulting in mitochondrial damage and the cleavage of downstream caspases 7, 14. Our results suggested that increased RIPK1 was observed in PHG tissues and that this upregulation of RIPK1 could also be induced and localized in mitochondria by TNF-α in the gastric epithelial cells. Mitochondria are highly dynamic organelles with high frequencies of fission and fusion events, and excessive mitochondrial fission always causes a collapse of the mitochondrial membrane potential and enhances mtROS production 23, 24. Drp1 is a key factor in mitochondrial fission and can be phosphorylated by RIPK1 at Ser616 to increase Drp1 translocation from the cytoplasm to the outer mitochondrial membrane 15, 20, where it interacts with abundant binding partners to mediate mitochondrial fission. We found that the changes in Drp1 and p-Drp1^S616^ levels presented a similar trend to that of RIPK1, FADD and caspase-8 (the FADDosome) in PHG, and a direct interaction among them was also confirmed. The enhanced direct interaction between Drp1 and Fis1 in mitochondria was further verified. Inhibition of mitochondrial fission by Mdivi-1 maintained mitochondrial Ca^2+^ homeostasis, attenuated mtROS production and mucosal injury in mice with PVL. These findings indicate that the FADDosome translocates to mitochondria to promote Drp1/Fis1-mediated mitochondrial fission. RIPK1 is a key factor in determining whether a cell lives or dies, and its ubiquitination is essential for survival signaling 10. Consistent with these findings, we also confirmed that FADD enhanced RIPK1 activity by inhibiting its ubiquitination and degradation.

Because the FADDosome aggravated mitochondrial dysfunction and mtROS generation in PHG, we wondered whether other mechanisms activated by this complex exist and may also increase mtROS generation in addition to mitochondrial fission. Multiple NOXs were modulated in PHG sections compared to the control tissues, and in particular, increased NOX2 levels were observed. NOX2 is involved in signal transduction and cell death 25, 47. The FADDosome was found to upregulate NOX2 expression by interacting with the TAK1/NF-κBp65 network and recruiting it to bind to the mitochondrial membrane to participate in inducing mitochondrial dysfunction and mtROS production in the epithelial cells of PHG. NOX2 promotes ROS production and can change mitochondrial morphology and activity, and Drp1 and NOX2 regulate each other and work together to induce NLRP3 inflammasome activation 37. Bax inhibitor 1 (BI1) inactivates the Syk-NOX2 pathway to subsequently repress Drp1-mediated mitochondrial fission and sustain mitochondrial function 48. NOX2 was also demonstrated to mediate oxidative stress and Drp1-mitochondrial fission after Japanese encephalitis viral infection 49. These studies suggest that NOX2-regulated mitochondrial fission by Drp1 is involved in several pathophysiological processes. In the present study, we also explored whether NOX2 is responsible for Drp1-mediated mitochondrial fission in PHG. NOX2 upregulation was observed in the gastric mucosa of PHG, and inhibition of NOX2 by GSK2795039 reduced the levels of 4-HNE and mtROS in the epithelial cells of the mice with PVL. Moreover, GSK2795039 decreased the upregulation of Drp1 and Fis1 but not that of FADD or caspase-8 in epithelial cells from mice with PVL, and it also alleviated the degree of mitochondrial fragmentation in epithelial cells from mice with PVL. However, Mdivi-1 did not influence the status of NOX2 in mice with PVL. These data demonstrate that NOX2, a downstream element of the FADDosome, enhances Drp1-dependent mitochondrial fission in PHG.

NOX2 is also intimately related to mitochondrial efficiency and glycolysis. NOX2 increases ROS levels, which in turn causes mitochondrial fission, reduces mitochondrial respiration and therefore promotes glycolysis 37. Some studies reveal that elevated NOX2 can induce HK2-dependent and 6-phosphofructo-2-kinase/fructose-2,6-bisphosphatase (PFKFB3)-dependent high glycolytic activity in glioblastoma multiforme (GBM) and acute myeloid leukemia (AML) cells, respectively 50, 51. Polarization toward M1 macrophages was dependent on NOX2 to increase the trafficking of glucose transporters to the membrane and consequently enhance glucose uptake for glycolysis. In our study, we found that the inhibition of NOX2 by GSK2795039 increased the production of ATP and reduced the levels of 4-HNE and mtROS in the gastric mucosa of mice with PVL. Moreover, the increase in LDHA and HK2 levels in the epithelial cells of mice with PVL was significantly decreased by GSK2795039. By detecting the OCR, we found that increased nonmitochondrial oxygen consumption, accompanied by reduced maximum respiration, occurred in the primary epithelial cells of mice with PVL, and this change was reversed by GSK2795039. GSK2795039 also repressed basal and compensatory glycolysis, as determined by ECAR analysis, in mice with PVL. Based on the abovementioned data, we revealed that NOX2, a downstream element of the FADDosome, contributed to the pathogenesis of PHG by enhancing mtROS production, regulating Drp1-dependent mitochondrial fission and influencing glycolytic activity.

Mitochondrial respiration and glycolysis are the two main ways of providing energy to mammalian cells. The FADDosome induced mitochondrial dysfunction and inhibited the intracellular generation of ATP in PHG, and increased glycolytic flux was observed in the PHG sections, as evidenced by increased HK2, HK3, LDHA, PKM, PFKL, PFKFB3 and PFKP levels. Nec-1 caused significant alterations in the cellular metabolic profile, including increased glucose and ATP generation as well as decreased lactate. Although aerobic glycolysis can also increase ATP levels to support energy supplementation in the tricarboxylic acid cycle, the total energy metabolism profile is affected by FADDosome-modulated mitochondrial dysfunction. A simple explanation is that multiple mechanisms contribute to alterations in glycolytic status and lactate secretion, which depend partly on mitochondrial fission and dysfunction and are associated with abnormal ATP production rates. Mitochondrial ATP generation and ROS production are intimately linked through the function of the ETC. Glycolysis supplies NADH and FADH_2_ to the ETC, each of which can donate a pair of electrons to the ETC. The predominant route of ROS production by the ETC is the premature leakage of electrons from complexes I, II and III to mediate the one-electron reduction of oxygen to superoxide (O_2_•-), which can then be dismutated to hydrogen peroxide (H_2_O_2_) to increase mtROS generation 42. ETC dysfunction always decreases mitochondrial energy production and enhances mtROS generation, which has been linked to the onset and development of various biological changes in organelles. In our study, we found that the levels of ETC complex subunits, including NDUFA9, SDHA, Cyt b, COX I and ATP5A, as well as the SOD activity and the GSH/GSSG ratio, were obviously decreased in PHG patients and mice with PVL but could be restored by Nec-1 or Mdivi-1 treatment in mice with PVL. These data suggest that ETC dysfunction and alterations in the activity of antioxidant enzymes (such as GSH and SOD) occur in PHG and contribute to the mtROS generation under altered glycolysis status.

In addition to being a glycolytic enzyme, PKM2 participates in additional cellular processes as a protein kinase, and the PKM2 isomeric form can act as a transcription factor to enhance the transactivation of genes encoding glucose transporters and glycolytic enzymes, including PDK1, LDHA, and Glut1, which are associated with cell proliferation, metastasis, apoptosis and inflammation 39-41. In our study, we detected the downstream proteins of PKM2 following *shFADD* transfection to analyze whether PKM2 is involved in FADDosome-regulated glycolysis in PHG, and we found that *FADD* knockdown by *shFADD* repressed LHDA upregulation in mice with PVL without affecting the levels of the isomeric forms of PKM2 (monomer, dimer or tetramer), Glut1 or PDK1. Moreover, *shFADD* decreased the levels of LHDA and Glut1 but did not alter the expression of PKM2 isomeric forms or PDK1 in TNF-α-treated GES-1 cells. These data suggested that PKM2-related signaling was not involved in FADDosome-regulated glycolysis in the PHG. Although the protein levels of PKM2 were increased in both PHG patients and mice with PVL, while the upregulation of PKM2 was not affected by Nec-1 treatment or *shFADD* transfection, which suggesting PKM2 may contribute to the pathogenesis of PHG by other signaling pathways rather than FADDosome network, and this underlying mechanism needs us to uncover in our future works.

FADDosome-regulated mitochondrial dysfunction increased mtROS production. Many studies have shown that mitochondrion is a platform for NLRP3 inflammasome activation, which requires mitochondrial Ca^2+^ uptake and mtROS accumulation 25, 42. MtROS is naturally produced as metabolic byproducts of aerobic mitochondrial metabolism under normal physiological conditions, and excessive mtROS causes oxidative damage to cellular proteins, lipids and nucleic acids to trigger NLRP3 inflammasome activation and pyroptosis 42, 52. The NLRP3 inflammasome is the molecule most closely associated with the initiation of pyroptosis. NLRP3 activation recruits ASC complexes and pro-caspase-1 to lead to autocleavage of caspase-1, which contributes to the cleavage and maturation of pro-IL-18 and pro-IL-1β and the cleavage of GSDMD (forming GSDMD-N). GSDMD-N then forms pores in the plasma membrane to enhance the secretion of IL-18 and IL-1β, resulting in pyroptosis. Excessive pyroptosis causes immoderate and continuous inflammatory responses that are involved in the occurrence of various diseases 26. The ROS scavenger Mito-TEMPO (MT) is a mitochondrion-targeted antioxidant that has strong antioxidant properties and accumulates several-fold within mitochondria to reverse Drp1-mediated mitophagy and mitochondrial fission by inhibiting superoxide production and mitigating mitochondrial oxidative stress 53, 54. Taken together, our results confirmed that pyroptotic elements, such as NLRP3, ASC, cleaved caspase-1, GSDMD-N, IL-1β and IL-18, were increased in PHG. Moreover, MT obviously relieved mucosal injury, enhanced epithelial cell viability, decreased NLRP3, cleaved caspase-1 and GSDMD-N levels, and decreased IL-1β and IL-18 production in mice with PVL without affecting the FADDosome in mice with PVL, which suggested that blockade of mtROS by MT provided protection against this epithelial NLRP3 inflammasome-mediated pyroptosis and mucosal injury in PHG. Furthermore, compared with those in *NLRP3*-KO mice, there was marked gastric injury with mucosal erosion and oxidative stress and enhanced NLRP3 inflammasome activation in PVL-treated *NLRP3*-WT mice. Fragmented mitochondrial morphology and disruption of membrane integrity with broken or absent cristae were aggravated in PVL-treated *NLRP3*-WT mice, although *NLRP3* deficiency partly rescued this mitochondrial damage, suggesting that mitochondrial dysfunction-induced NLRP3 inflammasome activation and pyroptosis could also act as negative feedback regulators of mitochondrial morphology and status. FADD and RIPK1 can activate caspase-8 and subsequently form a complex, causing GSDMD to be cleaved into GSDMD-N and eventually leading to pyroptosis 55, 56. In our study, epithelial NLRP3 inflammasome activation triggered mitochondrial GSDMD pore formation and the cleavage and activation of caspase-1, which converted pro-IL-1β and pro-IL-18 to their mature forms and eventually led to gastric epithelial pyroptosis and mucosal injury in PHG.

Various studies have revealed the contribution of caspase-8 catalytic activity to NLRP3 inflammasome activation, and caspase-8 may be directly involved in the cleavage of GSDMD and cause pyroptosis 8, 12, 44. However, we verified that the presence or absence of Z-VAD-FMK induced high amounts of polymerized ASC and NLRP3 and enhanced FADD and GSDMD-N in PVL-induced PHG mice without *shFADD*, while these effects were not altered in the epithelial fractions of mice with PHG treated with *shFADD*, regardless of Z-VAD-FMK treatment. The data indicated that the scaffolding function of caspase-8 but not its enzymatic activity was required in the FADDosome-regulated NLRP3 activation and GSDMD cleavage. Thus, understanding biochemical interactions allows researchers to find inhibitors or antagonists that target sequences specific to specific targets, decreasing the widespread side effects of other molecules or activators.

The formed FADD and caspase-8 complex is dissociated from the ligated receptor and recruits RIPK1 to form the FADDosome to support cellular death signaling 10, 11. In our previous studies, gastric mucosal epithelial apoptosis is an essential pathological characteristic in PHG 2, 4, 5. By utilizing pan-caspase inhibitor Z-VAD-FMK in our preliminary experiment, we found that blocking apoptosis did not completely alleviate the gastric mucosal injury in PHG, suggesting other types of programmed cell death may also participate in the pathogenesis of PHG. Caspase-8 represents the molecular switch that modulates apoptosis, necroptosis and pyroptosis 57, and caspase-8 inhibits mixed lineage kinase domain-like (MLKL)-dependent necroptosis by suppressing the function of RIPK1 or RIPK3 58, 59. Therefore, loss of caspase-8 or its catalytic activity leads to MLKL-dependent necroptosis. Under certain conditions, RIPK1 can initiate RIPK3-induced necroptosis instead of caspase-8-induced apoptosis. Necroptosis is highly dependent on RIPK3 as an essential part of the necroptotic machinery 60, 61. In our current study, accompany with FADD upregulation, caspase-8 was also highly expressed in similar gastric epithelial cells from PHG patients and mice with PVL, and direct enhancement of the interaction between FADD and caspase-8 was detected in the mice with PVL but not in the SO mice. Moreover, increased RIPK1 expression rather than RIPK3 expression was found in the gastric mucosal samples of mice with PVL and PHG patients compared with that in the corresponding control groups. The above information hints that the upregulation of caspase-8 in the gastric mucosal cells of PHG may restrict RIPK1/RIPK3/MLKL-dependent gastric epithelial necroptosis, and the underlying mechanism requires our further investigation and confirmation.

Activation of the FADDosome has the potential of triggering apoptosis, necroptosis and pyroptosis, and if either one or two of these pathways are blocked, the third pathway will still be able to result in cell death 58. Enhanced caspase-8 catalytic activity always leads to the cleavage of downstream caspases to induce apoptosis 44, 57. Besides caspases-mediated epithelial apoptosis in PHG from our previous studies, our current data revealed FADDosome could phosphorylate Drp1 by RIPK1 and enhance Drp1-mediated mitochondrial fission to result in mitochondrial dysfunction and excessive mtROS production, which caused oxidative damage to cellular proteins, lipids and nucleic acids to trigger NLRP3 inflammasome activation and pyroptosis. By the way, the scaffolding function of caspase-8 but not its enzymatic activity was found to be required in the FADDosome-regulated NLRP3 activation and GSDMD cleavage. RIPK1 and caspase-8 can directly bind to and drive NLRP3 activation 62, and FADD and caspase-8 can influence the NLRP3 inflammasome at the level of priming, activation, and post-assembly 6, 44. From these above mentioned, besides apoptosis, FADDosome-regulated pyroptosis was also an important way of mucosal epithelial death in PHG. Given the importance of the three types of cell death mechanisms (apoptosis, necroptosis and pyroptosis) in various pathologies, understanding the interplay between these pathways provides insights into how these modes of cell death are regulated to control the development of PHG.

## Conclusion

In conclusion, the FADDosome enhances mitochondrial dysfunction and influences the metabolic profile by altering glycolysis to increase mtROS production to drive NLRP3 inflammasome activation, and thus the activation of NLRP3 inflammasome results in gastric epithelial pyroptosis and mucosal injury in PHG, and this network provides a potential therapeutic target for PHG (**Figure 9H**).

## Supplementary Material

Supplementary figures.

## Abbreviations

AML: acute myeloid leukemia
ASC: apoptosis-associated speck-like protein
ATP5A: alpha subunit of ATP synthase
BI1: Bax inhibitor 1
CK18: cytokeratin 18
COX I: cyclooxygenase I
Cyt b: cytochrome b
DAPI: 4',6-diamidino-2-phenylindole dihydrochloride
DCFH-DA: 2,7-dichlorodihydrofluorescein diacetate
Drp1: dynamin-related protein 1
DRs: death receptors
ECAR: extracellular acidification rate
ECL: enhanced chemiluminescence
ETC: electron transport chain
FADD: Fas-associated protein with death domain
FADH_2_: flavin adenine dinucleotide
FasL: Fas ligand
FCCP: carbonyl cyanide-4 (trifluoromethoxy) phenylhydrazone
Fis1: fission protein 1
GBM: glioblastoma multiforme
GEV: gastroesophageal varices
Glut1: glucose transporter 1
GSH: glutathione
GSSG: glutathione disulfide
GSDMD: gasdermin D
GSDMD-N: N-terminal fragment of GSDMD
HBV: hepatitis B virus
H&E: hematoxylin and eosin
HIF-1: hypoxia-inducible factor 1
HK2: hexokinase-2
HK3: hexokinase-3
IFX: infliximab
HP: Helicobacter pylori
IF: immunofluorescence
IHC: immunohistochemical
IP: immunoprecipitation
LDHA: lactate dehydrogenase A
MAPKs: mitogen-activated protein kinases
MDA: malondialdehyde
Mdivi-1: mitochondrial division inhibitor 1
Mef: mitochondrial elongation factor
Mff: mitochondrial fission factor
Mfn1: mitofusin 1
Mfn2: mitofusin 2
Mid49: mitochondrial dynamics protein 49
Mid51: mitochondrial dynamics protein 51
MLKL: mixed lineage kinase domain-like
MT: Mito-TEMPO
mt 8-OHdG: mitochondrial 8-OHdG
mtROS: mitochondrial reactive oxygen species
NADH: nicotinamide adenine dinucleotide
NADPH: nicotinamide adenine dinucleotide phosphate
NDUFA9: NADH ubiquinone oxidoreductase subunit A9
Nec-1: necrostatin-1
NF-κBp65: nuclear factor-κBp65
NOX: nicotinamide adenine dinucleotide phosphate oxidase
OCR: oxygen consumption rate
OPA1: optic atrophy 1
ox-mtDNA: oxidized mitochondrial DNA
PDK1: pyruvate dehydrogenase kinase 1
PFKFB3: 6-phosphofructo-2-kinase fructose-2, 6-biphosphatase 3
PFKL: phosphofructokinase L
PFKP: phosphofructokinase P
PHT: portal hypertension
PHG: portal hypertensive gastropathy
PKM: pyruvate kinase M
PLS-DA: partial least squares discriminant analysis
PVL: portal vein ligation
RIPK1: receptor-interacting serine/threonine-protein kinase 1
Rot/AA: rotenone and antimycin A
SDHA: succinate dehydrogenase complex flavoprotein subunit A
SO: sham operation
SOD: superoxide dismutase
SPF: specific pathogen-free
TAK1: transforming growth factor-β-activated kinase 1
TEM: transmission electron microscopy
TNFR1: TNF-α/TNF receptor 1
Z-VAD-FMK: benzyloxycarbonyl-Val-Ala-Asp (OMe) fluoromethylketone
2-DG: 2-deoxyglucose
4-HNE: 4-hydroxynonenal
