# Supplementary material for: Mitochondrial Dysfunction by FADDosome Promotes Gastric Mucosal Injury in Portal Hypertensive Gastropathy
Source: Int J Biol Sci. 2024 Apr 29;20(7):2658–85. doi: 10.7150/ijbs.90835 (PMC11077381; doi:10.7150/ijbs.90835)
Supplement: Supplementary file 1 — Supplementary figures. [file ijbsv20p2658s1.pdf]

Supplementary materials

Supplementary Figures and Figure Legends

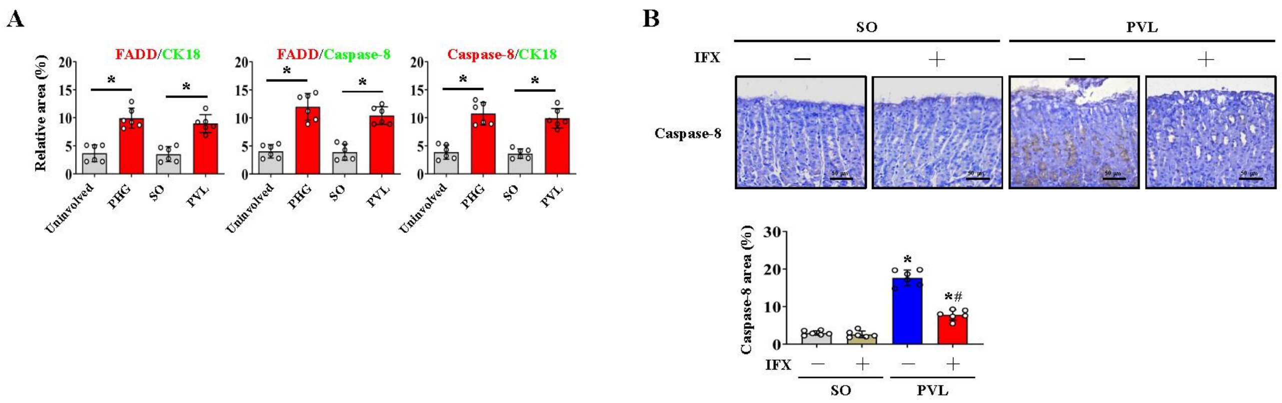

Supplementary Figure 1. FADD binds to caspase-8 in PHG, and IFX represses caspase-8 expression in mice with PVL. (A) The histograms show the area coexpressing FADD/CK18, FADD/caspase-8 or caspase-8/CK18 from Figure 1G to Figure 1I. n = 6 per group. *$P < 0.05$. (B) Immunohistochemical staining for caspase-8 (brown) showed that IFX repressed caspase-8 expression in mice with PVL. The caspase-8 area was also analyzed. *$P < 0.05$ versus SO mice; #$P < 0.05$ versus mice with PVL without IFX treatment.

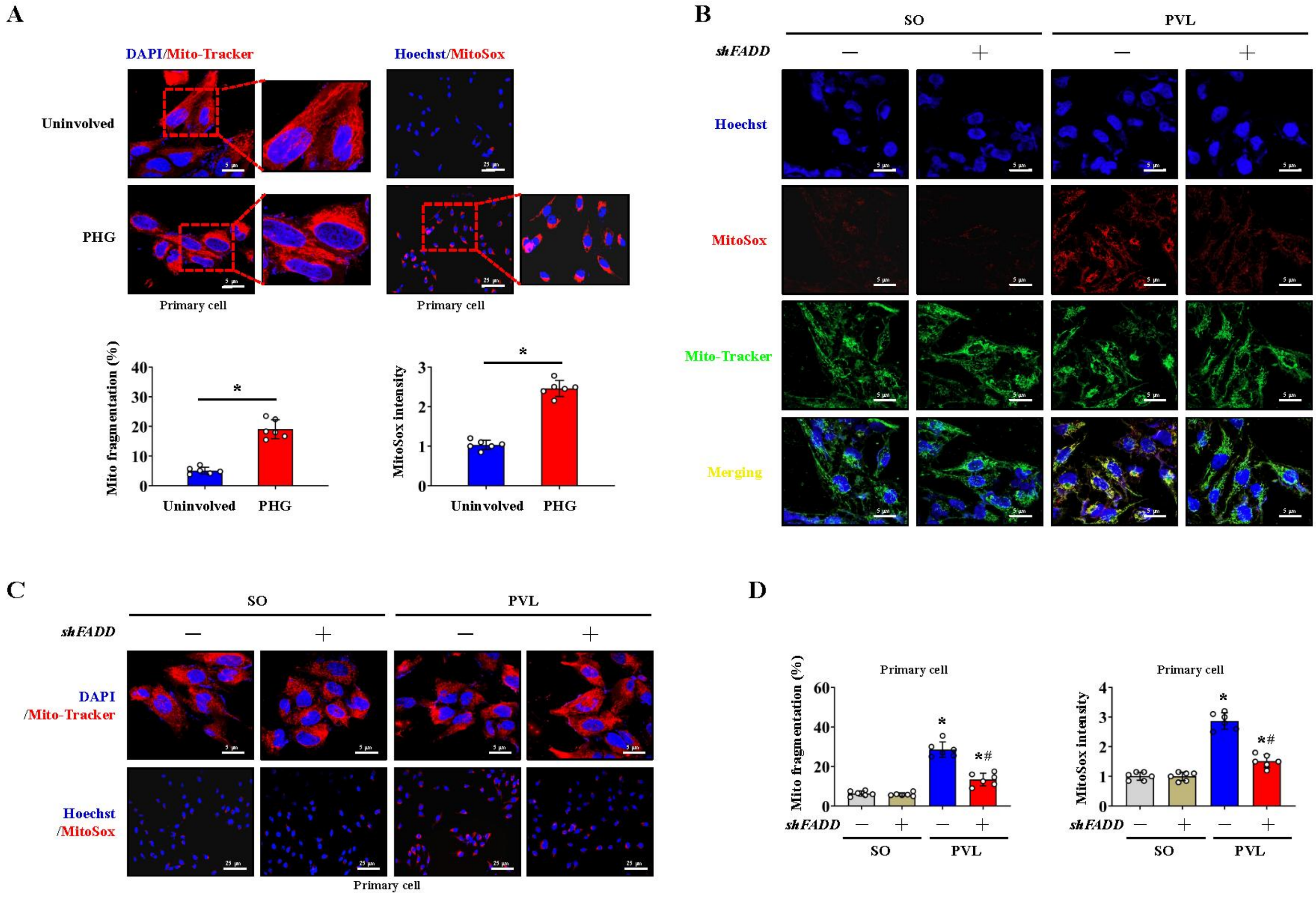

Supplementary Figure 2. FADD/caspase-8 regulates gastric epithelial mitochondrial fragmentation and ROS generation in PHG. (A) Mito-Tracker and MitoSox staining of primary epithelial cells from PHG patients and healthy volunteers (as Uninvolved) revealed enhanced mitochondrial fragmentation and ROS, and quantitative analysis of mitochondrial fragmentation index and mtROS levels was also performed. Nuclei (blue) were counterstained with DAPI or Hoechst. n = 6 per group; $*P < 0.05$. (B) Costaining of MitoSox (red) and Mito-Tracker (green) revealed that ROS were generated from mitochondria in the primary epithelial cells of mice with PVL, and *FADD* knockdown by *shFADD* attenuated this process. Nuclei (blue) were counterstained with Hoechst. (C) Mito-Tracker (red) and MitoSox (red) staining of mouse models with or without *shFADD* transfection revealed that *shFADD* attenuated mitochondrial fragmentation and ROS levels in mice with PVL. Nuclei (blue) were counterstained with DAPI or Hoechst. (D) Quantitative analysis of mitochondrial fragmentation and MitoSox intensity from (C) was presented. n = 6 in each group. $*P < 0.05$ versus SO mice; $\#P < 0.05$ versus mice with PVL without *shFADD* transfection.

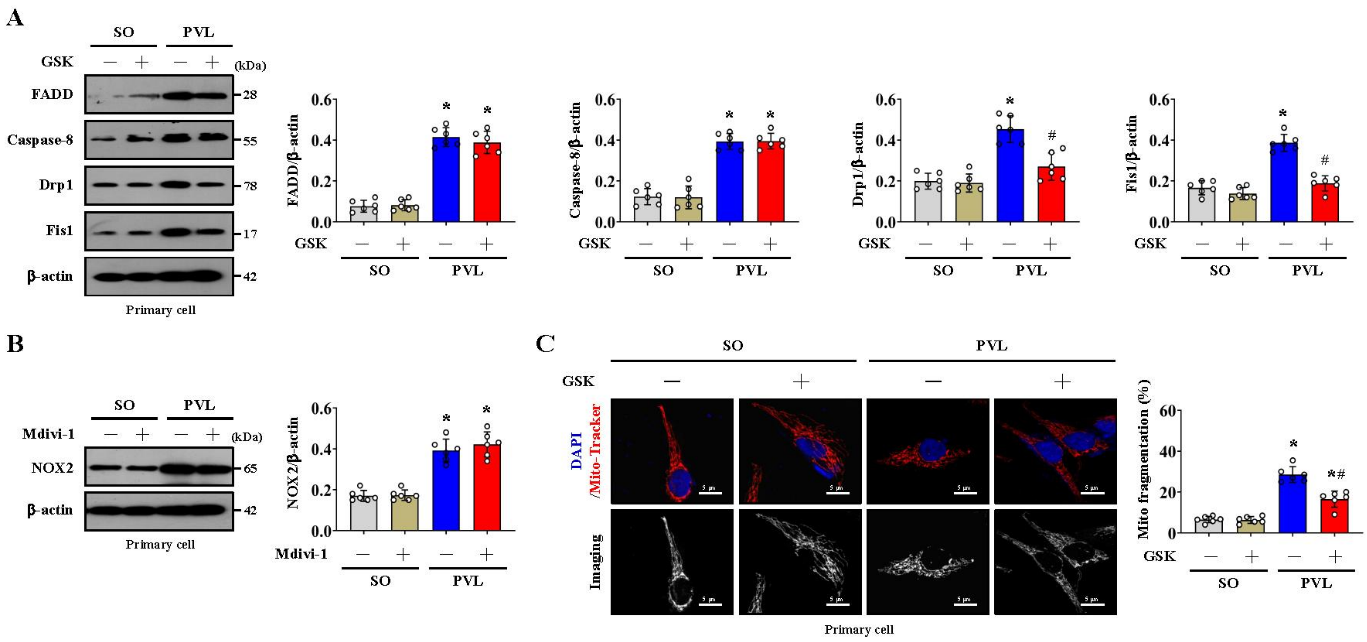

Supplementary Figure 3. Inhibition of NOX2 attenuates Drp1-dependent mitochondrial fission in PHG. (A) Representative western blotting for FADD, caspase-8, Drp1 and Fis1 showed that GSK2795039 (GSK, a NOX2 inhibitor) decreased the levels of Drp1 and Fis1 but not those of FADD or caspase-8 in epithelial cells from mice with PVL. The ratios of the normalized FADD/β-actin, caspase-8/β-actin, Drp1/β-actin and Fis1/β-actin densitometric units were also determined. n = 6 per group. **P* < 0.05 versus SO mice; #*P* < 0.05 versus mice with PVL not treated with GSK2795039. (B) Western blotting revealed that Mdivi-1 did not affect the levels of NOX2 in epithelial cells from mice with PVL. The ratio of densitometric units of the normalized NOX2/β-actin was determined. n = 6 per group. **P* < 0.05 versus SO mice. (C) Mito-Tracker (red) staining and mitochondrial imaging (white) of epithelial cells isolated from the mouse models revealed that GSK2795039 blocked mitochondrial fragmentation in mice with PVL. Nuclei (blue) were counterstained with DAPI. The quantitative analysis of the mitochondrial fragmentation was also presented. n = 6 per group. **P* < 0.05 versus SO mice; #*P* < 0.05 versus mice with PVL not treated with GSK2795039.

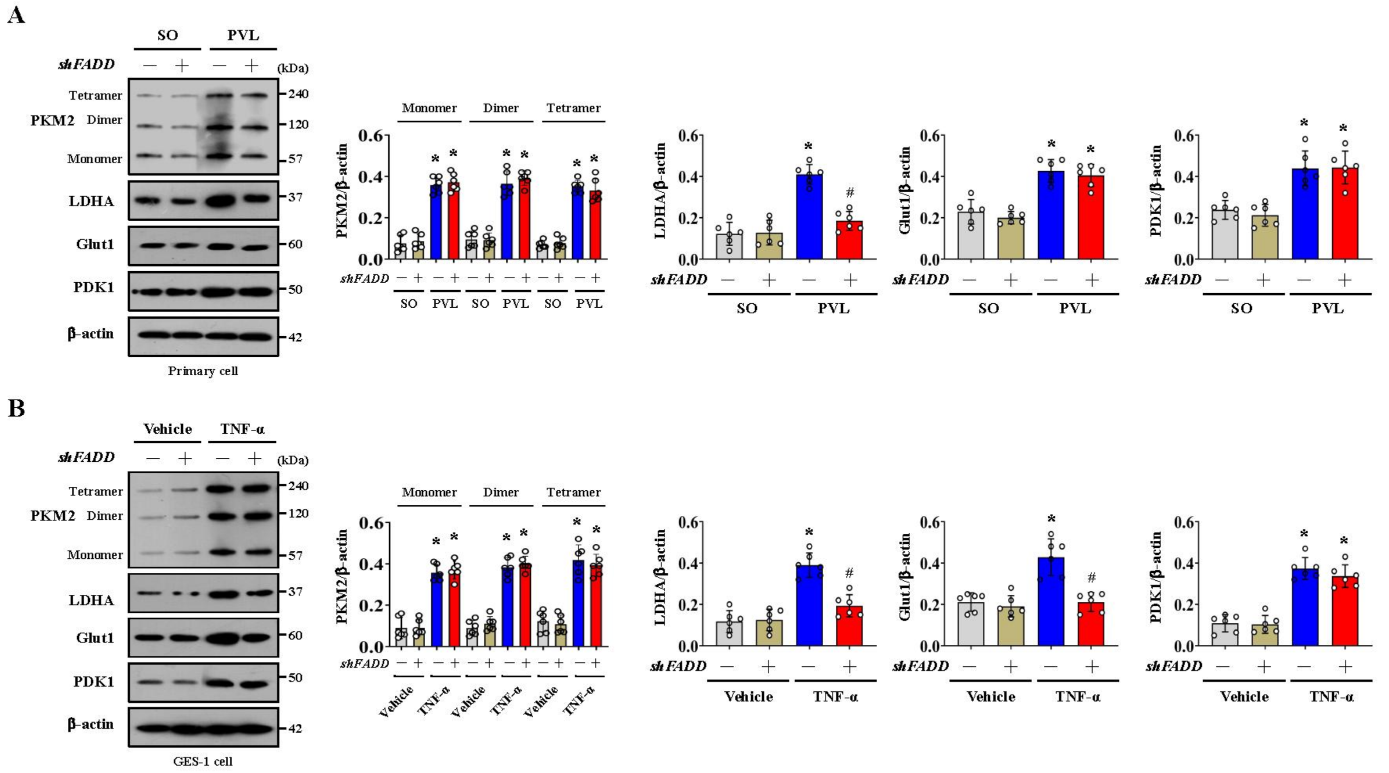

Supplementary Figure 4. ***FADD*** **knockdown has no effect on PKM2-related signaling in PHG.** (A) Western blotting for PKM2 (monomer, dimer and tetramer), LDHA, Glut1 and PDK1 in gastric epithelial cells from mouse models revealed that *FADD* knockdown by *shFADD* repressed LHDA upregulation in mice with PVL without affecting the levels of PKM2 isomeric forms (monomer, dimer and tetramer), Glut1 or PDK1. The ratios of densitometric units of the normalized PKM2/β-actin, LDHA/β-actin, Glut1/β-actin and PDK1/β-actin were also determined. n = 6 per group. *$P < 0.05$ versus SO mice; #$P < 0.05$ versus mice with PVL without *shFADD* transfection. (B) The levels of PKM2 isomeric forms (monomer, dimer and tetramer), LDHA, Glut1 and PDK1 in the indicated GES-1 cells determined by western blotting showed that *shFADD* repressed the upregulation of LHDA and Glut1 without affecting the expression of the isomeric forms of PKM2 (monomer, dimer and tetramer) or PDK1 in TNF-α-treated cells. The ratios of densitometric units of the normalized PKM2/β-actin, LDHA/β-actin, Glut1/β-actin and PDK1/β-actin were also presented. n = 6 per group. *$P < 0.05$ versus the vehicle group; #$P < 0.05$ versus TNF-α-treated cells without *shFADD* transfection.

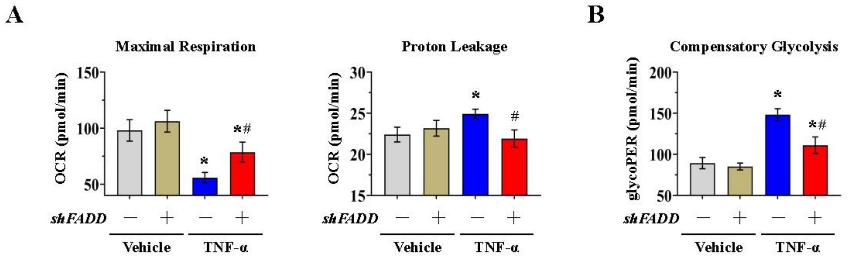

Supplementary Figure 5. FADD regulates the alteration of TNF-α-induced mitochondrial respiration and glycolysis. (A) OCR assays revealed that TNF-α treatment increased proton leakage and reduced maximum respiration in GES-1 cells, changes that were reversed by *FADD* knockdown (*shFADD* transfection). n = 6 in each group. **P* < 0.05 versus vehicle group; #*P* < 0.05 versus TNF-α-treated cells without *shFADD* transfection. (B) The ECAR assay showed that compensatory glycolysis was increased in response to TNF-α treatment and was rescued by *shFADD*. n = 6 in each group. **P* < 0.05 versus vehicle group; #*P* < 0.05 versus TNF-α-treated cells without *shFADD* transfection.

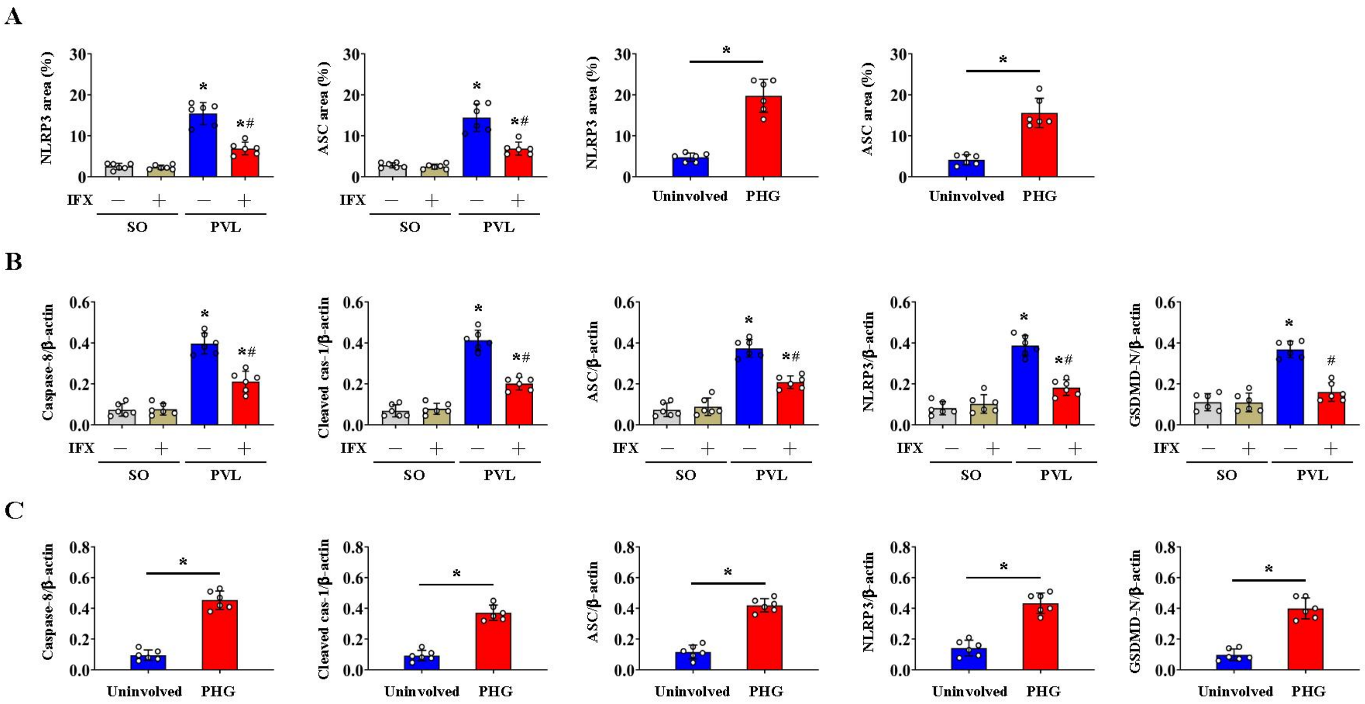

Supplementary Figure 6. NLRP3 inflammasome-mediated pyroptosis occurs in PHG, and IFX represses pyroptosis in mice with PVL. (A) The NLRP3 and ASC area analysis shown in Figure 8A revealed that NLRP3 inflammasome-related elements were induced in the PHG and IFX decreased that in the mice with PVL. n = 6 per group. *$P < 0.05$ versus SO mice or the uninvolved group; #$P < 0.05$ versus mice with PVL without IFX treatment. (B and C) The ratios of densitometric units of the normalized caspase-8/β-actin, cleaved caspase-1/β-actin, ASC/β-actin, NLRP3/β-actin and GSDMD-N/β-actin analyzed from Figure 8B revealed that the levels of pyroptotic elements (caspase-8, cleaved caspase-1, ASC, NLRP3 and GSDMD-N) were obviously increased in PHG patients and mice with PVL compared with their controls, while IFX downregulated those in mice with PVL. n = 6 per group. *$P < 0.05$ versus SO mice or the uninvolved group; #$P < 0.05$ versus mice with PVL without IFX treatment.

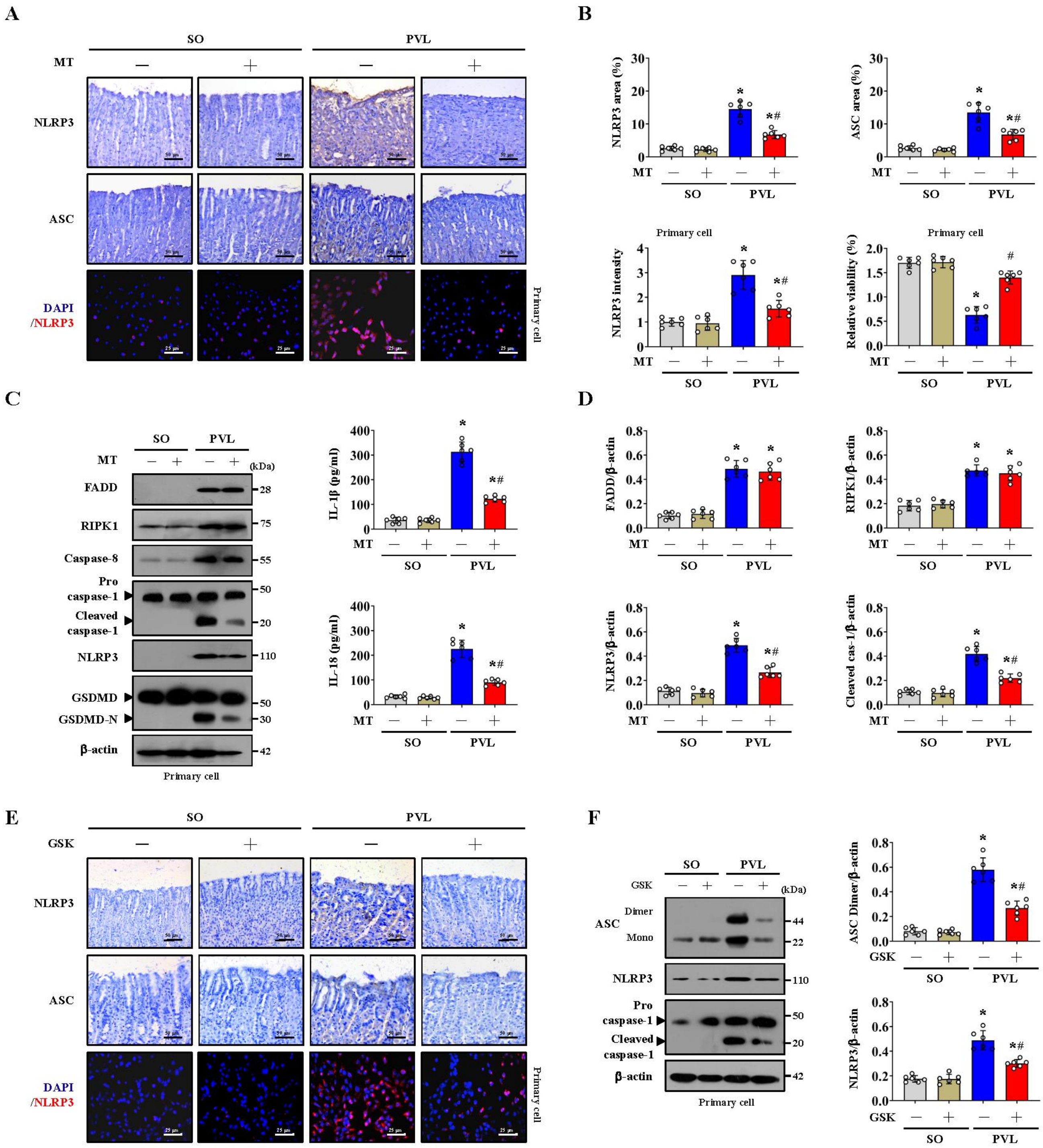

Supplementary Figure 7. Blockade of mitochondrial oxidative stress alleviates epithelial NLRP3 inflammasome activation and pyroptosis in PHG. (A) Representative photographs of NLRP3 and ASC IHC staining (brown) in mouse models and NLRP3 IF staining (red) in primary epithelial cells from SO mice and mice with PVL following treatment with the ROS scavenger Mito-TEMPO (MT). Nuclei (blue) were counterstained with DAPI for IF staining. (B) Quantitative analysis of the NLRP3 and ASC areas by IHC staining (upper panel). The NLRP3 intensity and cell viability (CCK-8 assay) of primary isolated epithelial cells (lower panel) suggested that MT reduced NLRP3 levels and recovered epithelial cell viability in mice with PVL. n = 6 per group. **P* < 0.05 versus SO mice; #*P* < 0.05 versus mice with PVL without MT treatment. (C) Representative western blotting (left panel) analysis of the expression of FADD, RIPK1,

caspase-8, procaspase-1, cleaved caspase-1, NLRP3, GSDMD and GSDMD-N and the medium concentrations of IL-1β or IL-18 (right panel) in primary epithelial cells from SO mice and mice with PVL following MT treatment. n = 6 per group. *P < 0.05 versus the SO group; #P < 0.05 versus the mice with PVL without MT treatment. (D) The ratio of densitometric units of the normalized FADD/β-actin, RIPK1/β-actin, NLRP3/β-actin and cleaved caspase-1/β-actin revealed that MT repressed NLRP3-related signaling without affecting the FADD/RIPK1 elements. n = 6 per group. *P < 0.05 versus the SO group; #P < 0.05 versus the mice with PVL without MT treatment. (E) Representative images of NLRP3 and ASC IHC staining (brown) in mouse models and NLRP3 IF staining (red) in primary cells isolated from SO mice and mice with PVL following treatment with the NOX2 inhibitor GSK2795039 (or not). Nuclei (blue) were stained with DAPI for IF staining. (F) Representative western blotting showing the expression of the ASC dimer, ASC monomer, NLRP3, procaspase-1 and cleaved caspase-1 in primary cells isolated from SO mice and mice with PVL treated with or without GSK2795039, and the ratios of densitometric units of the normalized ASC dimer/β-actin and NLRP3/β-actin were analyzed. n = 6 in each group. *P < 0.05 versus the SO group; #P < 0.05 versus the PVL group not treated with GSK2795039.

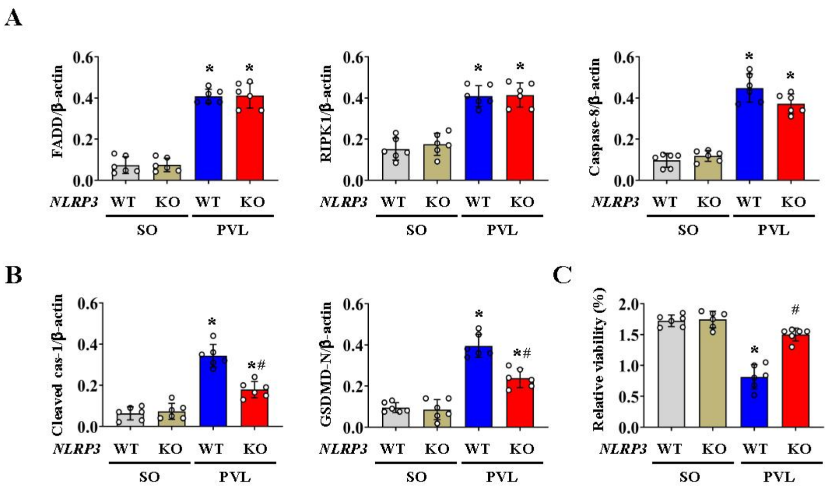

Supplementary Figure 8. *NLRP3* deficiency attenuates FADDosome-mediated gastric epithelial pyroptosis in mice with PVL. (A) The ratios of densitometric units of the normalized FADD/β-actin, RIPK1/β-actin, caspase-8/β-actin, Drp1/β-actin and Fis1/β-actin determined from Figure 9C revealed that *NLRP3* deficiency did not affect the expression of FADD, RIPK1, caspase-8, Drp1 or Fis1 in mice with PVL. n = 6 in each group. *$P < 0.05$ versus SO mice. (B) The ratio of densitometric units of the normalized cleaved caspase-1/β-actin and GSDMD-N/β-actin determined from Figure 9D revealed that *NLRP3* deficiency decreased the levels of cleaved caspase-1 and GSDMD-N in mice with PVL. n = 6 in each group. *$P < 0.05$ versus SO mice; #$P < 0.05$ versus PVL-treated *NLRP3*-WT mice. (C) The viability of primary cells from the indicated groups was analyzed by a CCK-8 assay, which showed that cell viability was markedly decreased in PVL-treated *NLRP3*-WT mice, and *NLRP3* deficiency restored that. n = 6 in each group. *$P < 0.05$ versus SO mice; #$P < 0.05$ versus PVL-treated *NLRP3*-WT mice.
